# CRISPR-Cas9 fusion to dominant-negative 53BP1 enhances HDR and inhibits NHEJ specifically at Cas9 target sites

**DOI:** 10.1038/s41467-019-10735-7

**Published:** 2019-06-28

**Authors:** Rajeswari Jayavaradhan, Devin M. Pillis, Michael Goodman, Fan Zhang, Yue Zhang, Paul R. Andreassen, Punam Malik

**Affiliations:** 1 Division of Experimental Hematology and Cancer Biology, Cancer and Blood Diseases Institute (CBDI), Cincinnati Children’s Hospital Medical Center (CCHMC), Cincinnati, OH 45229 USA; 2 Division of Allergy and Immunology, TriHealth, Cincinnati, OH 45040 USA; 3 Division of Hematology, CBDI, CCHMC, Cincinnati, OH 45229 USA; 4 Department of Pediatrics, University of Cincinnati College of Medicine, Cincinnati, OH 45229 USA

**Keywords:** Genetic engineering, Biotechnology, Molecular biology, CRISPR-Cas9 genome editing

## Abstract

Precise genome editing/correction of DNA double-strand breaks (DSBs) induced by CRISPR-Cas9 by homology-dependent repair (HDR) is limited by the competing error-prone non-homologous end-joining (NHEJ) DNA repair pathway. Here, we define a safer and efficient system that promotes HDR-based precise genome editing, while reducing NHEJ locally, only at CRISPR-Cas9-induced DSBs. We fused a dominant-negative mutant of 53BP1, DN1S, to Cas9 nucleases, and the resulting Cas9-DN1S fusion proteins significantly block NHEJ events specifically at Cas9 cut sites and improve HDR frequency; HDR frequency reached 86% in K562 cells. Cas9-DN1S protein maintains this effect in different human cell types, including leukocyte adhesion deficiency (LAD) patient-derived immortalized B lymphocytes, where nearly 70% of alleles were repaired by HDR and 7% by NHEJ. Our CRISPR-Cas9-DN1S system is clinically relevant to improve the efficiencies of precise gene correction/insertion, significantly reducing error-prone NHEJ events at the nuclease cleavage site, while avoiding the unwanted effects of global NHEJ inhibition.

## Abstract (web summary)

Global inhibition of NHEJ factors has been one strategy to improve CRISPR-Cas9 mediated HDR. Here the authors fuse a dominant-negative mutant of 53BP1 to Cas9 to enhance HDR frequency, reduce NHEJ specifically at the Cas9 cut sites, and reduce the toxicity associated with global NHEJ inhibition.

## Introduction

The CRISPR-Cas9 system allows for precise genome editing/correction by exploiting cellular homology-dependent repair (HDR)^1,2^. However, HDR efficiency is limited by competing and far more efficient double-strand break (DSB) repair pathways, primarily error-prone non-homologous end-joining (NHEJ)^3^. Global inhibition of key NHEJ factors has been the most studied strategy to improve HDR efficiency^4,5^. However, permanent inhibition of NHEJ factors results in bone marrow failure, stem cell ageing, and cancer susceptibility^6–11^ and temporary inhibition may also have deleterious effects on the genome.

In order to enhance genome editing by HDR, different approaches have been developed, including (i) cell synchronization in S/G2 phases^12^ or fusion of Cas9 to the Geminin degron to regulate Cas9 expression in the S-phase of cell cycle^13,14^ and (ii) overall inhibition of cellular NHEJ repair (global NHEJ inhibition) by inhibiting or depleting NHEJ proteins DNA Ligase IV, DNA-dependent protein kinase catalytic subunit (DNA-PKcs) and 53BP1^4,15,16^ or overexpression of DNA repair proteins such as RAD52 along with mouse dominant negative (mdn) 53BP1^17,18^. These treatments, albeit temporarily, block the overall cellular DNA repair which occurs from naturally occurring DSBs or DNA replication. Of these, transient inhibition of key NHEJ factors such as Ku70, DNA ligase IV, or DNA-PKcs, either by small molecules, shRNA, or proteolytic degradation, has been most widely used^4,5,16^.

Given the importance of NHEJ in genome maintenance, transient global NHEJ inhibition strategies may have adverse consequences on genome integrity. Indeed, permanent global inhibition of NHEJ factors such as DNA ligase IV and DNA-PKcs in humans results in severe combined immune deficiencies, pancytopenia and growth retardation^19^. Specifically, NHEJ deficiency in murine hematopoietic stem cells (HSC) is associated with bone marrow failure^9,10^, loss of ‘stemness’, ageing^20,21^, and cancer^11^. Here, we hypothesized that if error-prone NHEJ repair was specifically inhibited only at the DSB generated by Cas9, HDR efficiency would be enhanced, without compromising genome integrity, thereby promoting cellular homeostasis, and clinical safety.

A key regulator of the choice between NHEJ and HDR is tumor suppressor p53-binding protein 1 (53BP1)^22^. 53BP1 is a pro-NHEJ factor which limits HDR by blocking DNA end resection, and also by inhibiting BRCA1 recruitment to DSB sites^22–24^. Recent studies have shown that global inhibition of 53BP1 by a ubiquitin variant significantly improves Cas9-mediated HDR frequency in non-hematopoietic and hematopoietic cells with single-strand oligonucleotide delivery or double-strand donor in AAV^15^. However, global overexpression of mdn53BP1 via plasmid transfections did not improve HDR, unless combined with a Rad52 expression plasmid (a protein involved in transcription-associated HDR^25^) in a HEK-293 reporter cell line^17^.

The 53BP1 adapter protein is recruited to specific histone marks at sites of DSBs via a minimal focus forming region (FFR). The FFR includes the following conserved domains: an oligomerization domain (OD), a glycine-arginine rich (GAR) motif, a Tudor domain, and an adjacent ubiquitin-dependent recruitment (UDR) motif^26^. The Tudor domain mediates interactions with histone H4 dimethylated at K20^23^. Domains of 53BP1 that are outside this region, towards the N-terminus and tandem C-terminal BRCT repeats, recruit key effectors involved in NHEJ, such as RIF1-PTIP and EXPAND, respectively^27^.

We generated a dominant negative version of 53BP1 (DN1S) that suppresses the accumulation of endogenous 53BP1 and downstream NHEJ proteins at sites of DNA damage, while upregulating the recruitment of the BRCA1 HDR protein. We demonstrate that upon fusion of DN1S to Cas9, Cas9-DN1S is recruited in a Cas9/gRNA-specific manner to locally inhibit NHEJ at Cas9-target sites, while promoting an increase in HDR, and does not globally affect NHEJ, thereby improving cell viability. Therefore, Cas9-DN1S provides a safer alternative for boosting HDR-based precise genome editing events by specifically reducing NHEJ events at Cas9-induced breaks.

## Results

### Identification of an HDR-enhancing fragment of 53BP1

**Fig. 1:**
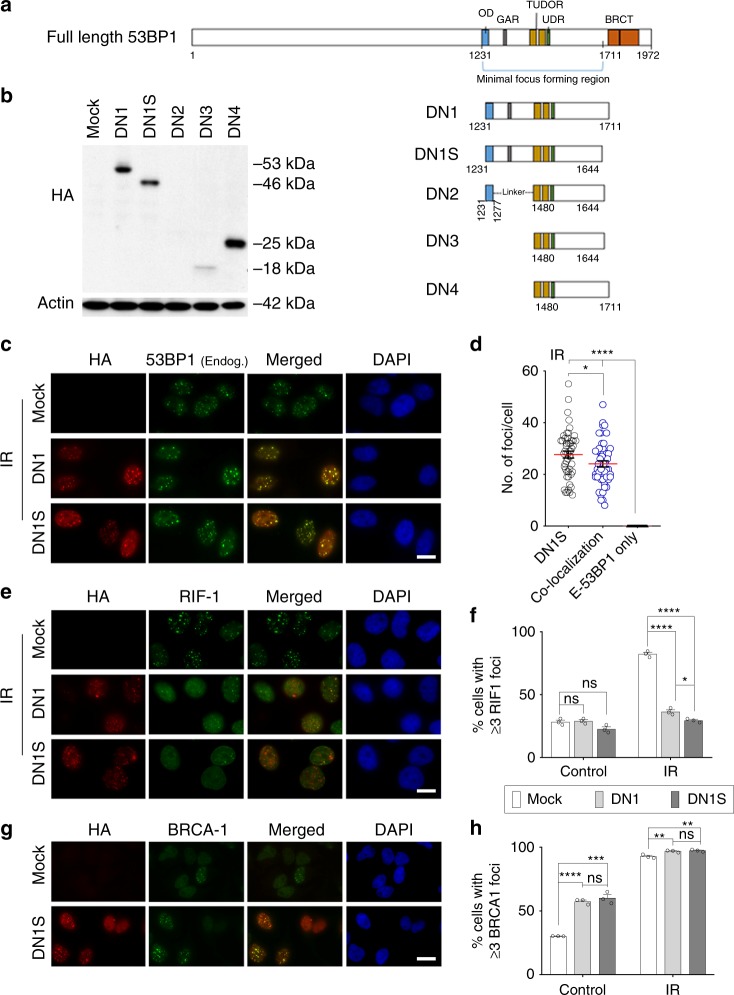
Identification of an HDR-enhancing fragment of 53BP1. **a** Schematic diagram of full-length human 53BP1 protein showing the minimal focus forming region including the oligomerization domain (OD), a glycine-arginine rich (GAR) motif, a tandem Tudor domain, and the ubiquitin-dependent recruitment (UDR) motif. BRCT repeats at the extreme C-terminus are present. **b** Different truncated 53BP1 proteins, DN1, DN1S, DN2, DN3, and DN4, were tested for their relative expression by western blot using anti-HA antibody in HeLa cells transduced with empty vector (mock) or lentiviral vectors encoding HA-tagged 53BP1 fragments DN1, DN1S, DN2, DN3, or DN4. Actin is shown as a loading control. **c**, **e**, **g** Representative immunofluorescence (IF) images of HA-tagged DN1 and DN1S at nuclear foci. Cells were either exposed to 2 Gy IR or no IR (control) and fixed 2 h later. Scale bars represent 20 μm. **c** Representative IF images showing co-localization of HA-tagged DN1 or DN1S with endogenous 53BP1 foci. **d** Number (no.) of HA+ (DN1S) foci, co-localized HA+/endogenous 53BP1+ (co-localization) foci, or endogenous 53BP1+ (E-53BP1) only foci per IR-treated HeLa cell expressing DN1S. Individual quantifications of foci from 50 cells in each group are shown. Red lines are drawn at the mean number of foci per positive cell. **e** Representative IF images showing HA-tagged DN1 or DN1S and RIF1 recruitment to IR-induced DNA repair foci. **f** Quantification of cells with ≥3 RIF1 foci in control cells or IR cells with DN1, DN1S or without vector (mock). **g** Representative IF images showing HA-tagged DN1S and BRCA1 recruitment to DNA repair foci in control cells. **h** Quantification of the number of cells with ≥3 BRCA1 foci in control cells or IR cells, with DN1, DN1S or without vector (mock). In panels **f** and **g**, data are presented as the mean ± SEM of counts of 150 cells each, from three independent fields, and black circles indicate individual counts. Statistics for panels **d**, **f**, and **g**: ANOVA. ns indicates not significant, *p < 0.05, ***p < 0.001, and ****p < 0.0001. Source data is available in Source Data file

We screened domains of human 53BP1 protein to make a reasonably small-sized dominant negative (DN) version for fusion with Cas9. We generated several putative DN human 53BP1 constructs, by removing regions of 53BP1 that bind RIF1, PTIP, and EXPAND (Fig. 1a). These proteins normally promote NHEJ at the expense of HDR by inhibiting DNA end resection, leading to recruitment of key effectors of NHEJ such as Ku70 and Ku80. We designed five different HA-tagged DN 53BP1 fragments to be delivered by lentiviral vectors: DN1 and DN1S (a shorter version of DN1), which contain the FFR; DN2, which excludes the GAR motif but retains the OD domain via a linker to the remaining FFR; DN3 and DN4, which exclude both the OD domain and the GAR motif from the FFR. At equivalent lentiviral transduction efficiency, DN1, DN1S, and DN4 showed robust expression, while DN2 and DN3 showed reduced expression of the recombinant 53BP1 proteins in HeLa cells (Fig. 1b and Supplementary Fig. 1a). Notably, DN1, DN1S, and DN2 localized to nuclear foci, in support of a potential dominant activity, whereas DN3 and DN4 only showed diffuse expression (Supplementary Fig. 1b). Thus, further studies were done with DN1 and DN1S, which both showed robust expression and localization to nuclear foci.

### DN1 and DN1S show dominant negative activity

To determine whether DN1 or DN1S has dominant activity by functionally competing with endogenous 53BP1, we stably expressed HA-tagged DN1 or DN1S in HeLa cells and co-stained for HA and endogenous 53BP1. We found that when expressed at modest levels, DN1 or DN1S largely co-localize with endogenous 53BP1 at irradiation induced foci (IRIF) (Fig. 1c). When expressed at higher levels, DN1S completely prevented endogenous 53BP1 localization to IRIF (Supplementary Fig. 1c). Notably, while DN1S IRIF could be observed in the absence of endogenous 53BP1 IRIF, there were no IRIF that were only positive for endogenous 53BP1. This suggests that DN1S actively competed with endogenous 53BP1 for binding to damaged chromatin (Fig. 1c, d). After localization to damaged chromatin, we wanted to assess if the dominant negative activity of DN1S inhibits the recruitment of downstream NHEJ proteins while improving the recruitment of HDR proteins to DNA repair foci. We observed a dramatic decrease in the recruitment of RIF1 (which promotes recruitment of other NHEJ proteins upon binding 53BP1) to IRIF (Fig. 1e, f) and a significant increase in the recruitment of BRCA1 (a key protein in the HDR pathway) to DNA repair foci or IRIF (Fig. 1g, h) in cells that stably express DN1S, as compared to mock (empty vector transduced) cells. Further, we observed an increase in levels of cells containing DN1S or DN1 that display BRCA1 foci in S/G2 phase, but an even greater and highly significant increase in BRCA1 foci in cells in the G0/G1 phase (without affecting the cell cycle) (Supplementary Fig. 2). Thus, DN1S and DN1 fragments are devoid of the domains involved in 53BP1-mediated repression of BRCA1 recruitment to foci in G1 cells^28^. We also observed that DN1S expression increased the number of γH2AX foci in non-irradiated cells, compared to mock cells. The presence of more γH2AX foci indirectly shows that DN1S acts to reduce the cellular NHEJ-mediated DSB repair and thereby increase the levels of unrepaired DNA damage (Supplementary Fig. 3a, b). Taken together, DN1S functions as a dominant negative 53BP1 protein, by co-localizing with or displacing endogenous 53BP1, and by reducing and increasing the recruitment of key NHEJ and HDR proteins, respectively, to DNA repair foci.

### Fusion to Cas9 confers increased specificity to DN1S

**Fig. 2:**
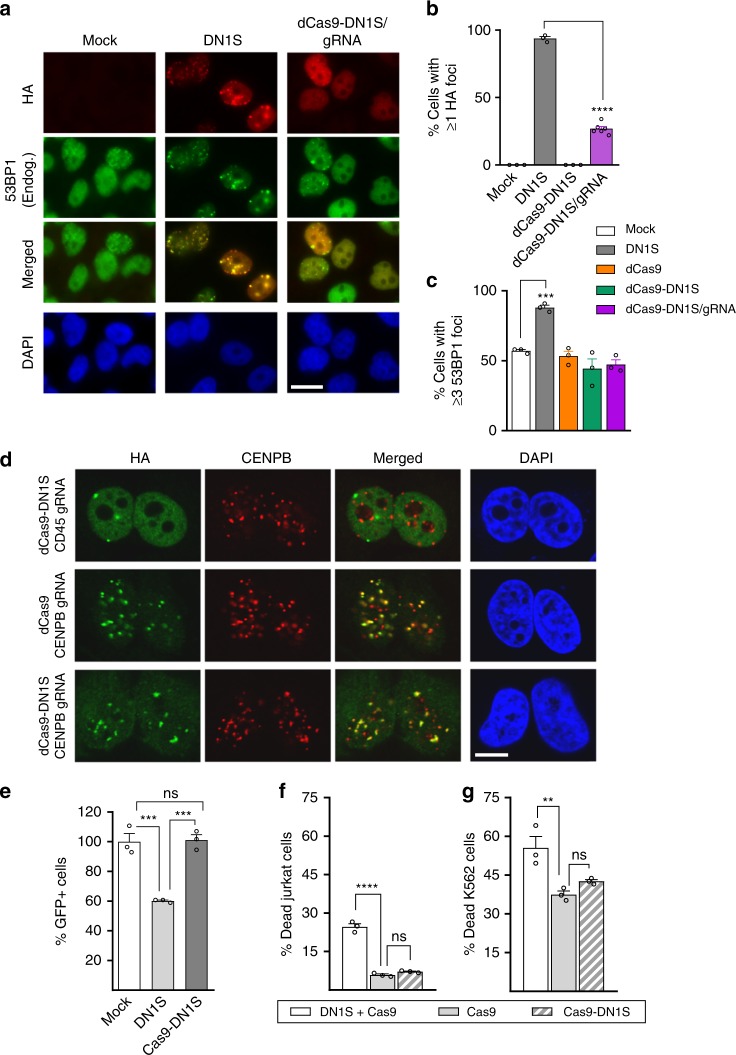
Cas9-DN1S blocks NHEJ locally, reducing toxicity associated with global NHEJ inhibition. **a** Representative immunofluorescence (IF) images showing HA-tagged DN1S or dCas9-DN1S/gRNA and endogenous 53BP1 recruitment to DNA repair foci. Scale bar represents 20 μm. **b** Quantification of the number of cells with ≥1 HA+ (DN1S) foci. **c** Quantification of the number of HeLa cells with ≥3 53BP1+ foci transduced with lentivirus for the following: mock (empty vector), DN1S, dCas9, dCas9-DN1S, and dCas9-DN1S/gRNA. **b**, **c** the data are presented as the mean ± SEM of three counts of 150 cells each from independent fields, except dCas9-DN1S/gRNA in **b**, which shows six counts of 150 cells from independent fields. Black circles indicate individual counts. Statistics: ANOVA, ***p < 0.001, ****p < 0.0001. **d** Representative IF images showing co-localization of dCas9 or dCas9-DN1S with CENPB gRNA to CENPB in centromeres. dCas9-DN1S with CD45 gRNA was used as a control. Scale bar represents 10 μm. **e** GFP+ HeLa cells with stable integration of the EJ5-GFP NHEJ reporter, after exposure to lentiviral constructs expressing DN1S or Cas9-DN1S, and lipofection with the I-SceI plasmid. I-SceI induced DSB repair via NHEJ at defined sites in the reporter allows for GFP expression. GFP+ cells were quantified by flow cytometry and data normalized to GFP+ cells in mock controls. Viability of (**f**) Jurkat cells and (**g**) K562 cells 48 h after DN1S or empty vector transduction, and 24 h after Cas9 or Cas9-DN1S RNP electroporation with AAV donor template delivery. Viability was quantified by flow cytometry using eFluor 780 fixable viability dye. **a**–**e** Cas9 denotes SpCas9. **f**–**g** Cas9 denotes SaCas9; **e**–**g** data are presented as the mean ± SEM of three independent transfections or electroporations. Black circles indicate individual data points. Statistics: ANOVA. ns indicates not significant, **p < 0.01, ***p < 0.001, ****p < 0.0001. Source data is available in Source Data file

To inhibit NHEJ only at Cas9-induced DSBs, and thereby prevent global cellular inhibition of NHEJ, we fused SpCas9 to DN1S, DN1 and DN2 (which also localized to nuclear foci) and its longer version, DN2L (Supplementary Fig. 4a). Fusion to Cas9 appeared to stabilize DN2 expression somewhat (Fig. 1b and Supplementary Fig. 4b), but we selected Cas9-DN1S for further study because it displayed the strongest expression. To validate that our modified CRISPR/Cas9-DN1S system specifically inhibits NHEJ at the Cas9/gRNA target site, and that DN1S does not mis-localize Cas9 to other naturally occurring cellular DNA repair foci, we fused DN1S to the catalytically dead Cas9 (dCas9) and performed functional studies. Here, dCas9 will remain at the target site in the genome, tethered via the gRNA, allowing visualization of the dCas9-DN1S fusion at foci, unlike the catalytically active nuclease, which may leave the target genomic locus after inducing the DSB. HA-tagged dCas9-DN1S fusion, with or without gRNA specific to a sequence in the *PTPRC* (*CD45*) gene (Supplementary Table 1), or HA-tagged DN1S alone were stably expressed in HeLa cells via lentiviral vectors. While still displaying some co-localization with endogenous 53BP1 (Fig. 2a and Supplementary Fig. 5b), as compared to DN1S alone, the dCas9-DN1S fusion protein with gRNA showed a significant reduction in the number of cells that had one or more DNA repair foci that co-localize with endogenous 53BP1 (Fig. 2a). Most importantly, cells expressing dCas9-DN1S without the gRNA behaved like mock cells, with no localization of dCas9-DN1S to any cellular nuclear foci (Fig. 2b and Supplementary Fig. 5a). However, the fact that dCas9-DN1S alone does not form foci or accumulate in nuclei may be attributed, at least in part, to decreased stability in the absence of a gRNA (Supplementary Fig. 5c). Notably, unlike cells expressing DN1S alone, the levels of endogenous 53BP1, γH2AX, BRCA1 and Rad51 foci in cells expressing dCas9-DN1S with CD45 gRNA or dCas9-DN1S with no gRNA were similar to cells expressing dCas9 with no gRNA or the empty vector (mock) control (Fig. 2c, Supplementary Fig. 6). Taken together, these data suggest that fusion to CRISPR/Cas9 confers specificity on DN1S, and that CRISPR/Cas9-DN1S does not displace endogenous 53BP1 by globally localizing to cellular DNA repair foci, thereby restricting the disruptive effects of DN1S on DNA damage response proteins.

Next, to rigorously test whether HA-tagged dCas9 or dCas9-DN1S is specifically targeted by gRNA, we utilized a gRNA against the CENPB box^29^, for which there are many repeats at centromeres. Indeed, we found that CENPB box gRNA drives co-localization of dCas9 and dCas9-DN1S with centromeres visualized by immunofluorescence microscopy (IF) using CENPB antibody (Fig. 2d). Because the gRNA is specific for only a subset of the CENPB box motifs, dCas9 and dCas9-DN1S do not co-localize with all centromeres. However, in the presence of CENPB box gRNA, none of the HA foci fail to co-localize with CENPB, so the patterns of HA and CENPB are strikingly similar. In contrast, gRNA to the CD45 locus, which is present only once per allele, does not show co-localization of dCas9-DN1S with centromeres (CENPB). Thus, the gRNA largely restricts dCas9-DN1S localization by targeting it to specific sites.

### Cas9-DN1S does not globally inhibit NHEJ

To confirm that functionally, CRISPR-Cas9-DN1S does not globally affect cellular NHEJ, we used an EJ5 NHEJ-reporter HeLa cell line, in which the promoter is separated from GFP cDNA by the puromycin-resistance gene (puro). Puro is flanked by I-SceI recognition sequences. Excision of puro by I-SceI and NHEJ repair results in GFP expression^30^. We transduced EJ5 HeLa cells with lentiviruses carrying untethered DN1S or Cas9-DN1S, or mock controls, both carrying the tNGFR reporter in addition. Transfection with the I-SceI plasmid significantly reduced the number of GFP+ cells with DN1S alone, unlike Cas9-DN1S and mock HeLa cells that had similar numbers of GFP+ cells. (Fig. 2e). This shows that the fusion protein does not non-specifically alter NHEJ repair, while DN1S alone does globally affect cellular NHEJ.

### Specifically localized Cas9-DN1S improves cell viability

Next, we tested the HDR of untethered DN1S in hematopoietic cells. We transduced Jurkat T cells and K562 myeloid cells with either DN1S lentivirus or empty vector controls, waited 24 h to allow expression of DN1S, and then electroporated the DN1S group of cells with Cas9 ribonucleoprotein (RNP), and the empty vector group of cells with either Cas9 RNP or Cas9-DN1S RNP. Cells were kept in bulk culture after electroporation for two weeks to assess HDR. We observed a significant increase in Jurkat and K562 cell death within 24 h in DN1S + Cas9 as compared to Cas9 and Cas9-DN1S cultures (Fig. 2f, g, Supplementary Fig. 7a). Although viability continued to be significantly lower for DN1S + Cas9 for 72 h in culture (shown in Supplementary Fig. 7b), only 24 h viability data is comparable across the groups, since RNP should not be present in all three groups after that period^31^, but DN1S, integrated from the lentivirus would continue to express in the DN1S + Cas9 cells. When HDR was assessed at 2 weeks, the untethered DN1S improved HDR to the same efficiency as Cas9-DN1S fusion protein in both K562 and Jurkat cells (Supplementary Fig. 7c).

As another functional test, we also performed a colony forming assay in HeLa cells, in response to ionizing radiation (IR). NHEJ-deficient cells are much more sensitive to IR than normal cells, due to their inability to repair IR-induced DNA damage via NHEJ, and this defective DNA repair can be quantified using a colony growth assay^32^. We selected HeLa cells that stably expressed DN1S without Cas9 fusion, Cas9-DN1S with gRNA, Cas9 with gRNA, or the empty vector control, and subjected them to increasing doses of IR, followed by plating cells and enumerating colony formation. HeLa cells with global downregulation/inhibition of NHEJ, using either a shRNA to 53BP1 or the DNA-PKcs inhibitor NU7441, were positive controls that resulted in significant cellular toxicity with increasing doses of IR (significant reduction in number of HeLa cell colonies), as compared to empty vector or scrambled shRNA controls (Supplementary Fig. 8a). The overexpression of DN1S without Cas9 fusion also reduced the number of colonies at high IR doses, since untethered DN1S globally inhibits NHEJ by displacing endogenous 53BP1 from cellular DNA repair foci. However, the Cas9-DN1S fusion protein with gRNA, like Cas9 protein with gRNA, showed no significant reduction in colony formation (Supplementary Fig. 8a). A similar lack of functional toxicity was seen with dCas9-DN1S gRNA complex (Supplementary Fig. 8b).

Taken together, unlike untethered DN1S, or global knock-down of 53BP1, or inhibition of DNA-PKcs, all of which reduce cellular NHEJ, Cas9-DN1S fusion protein restricts the global effects of DN1S on NHEJ and cell viability.

### Cas9-DN1S stimulates HDR in multiple cell lines

After establishing that CRISPR/Cas9-DN1S did not globally locate to cellular DNA repair foci or inhibit global cellular NHEJ, we tested its ability to improve HDR frequency and inhibit NHEJ events at Cas9-induced DSBs in the presence of an exogenous HDR donor template. We generated a previously reported human HEK293 traffic light reporter (TLR) system^5^, where NHEJ events at the *Rosa26* locus (the integrated TLR cassette) lead to red fluorescent cells (RFP+), and HDR events yield green fluorescent cells (Venus+). We targeted the TLR cassette to the *AAVS1* ‘safe harbor’ location in human 293T cells. In the TLR 293T cells, we found that CRISPR/Cas9-DN fusions remarkably reduced NHEJ repair, and increased HDR by 3-fold with SpCas9-DN1 or SpCas9-DN1S fusion constructs (Supplementary Fig. 9a). SpCas9-DN2 or SpCas9-DN2L also significantly reduced NHEJ at the Cas9 cut sites, but did not improve HDR efficiency, suggesting that the GAR motif and the amino acids in front of the Tudor domain are likely important for the HDR effect. We therefore used the Cas9-DN1S fusion for subsequent HDR experiments. We further investigated the optimal orientation of the DN1S (N-terminus or C-terminus fusion to Cas9) and different linkers that fuse DN1S to Cas9 to find a construct with the highest HDR to NHEJ ratio. While there were slight variations in cutting efficiency with the different epitope tags, the Flag-SpCas9-TGS linker-DN1S showed nearly 7-fold higher HDR efficiency and significantly reduced NHEJ-mediated repair when compared to Flag-SpCas9 (Supplementary Fig. 9b), and had the highest overall ratio of HDR:NHEJ ratio (6-fold) relative to Flag-SpCas9. The Cas9-TGS linker-DN1S cassette was used in all subsequent HDR experiments.

**Fig. 3:**
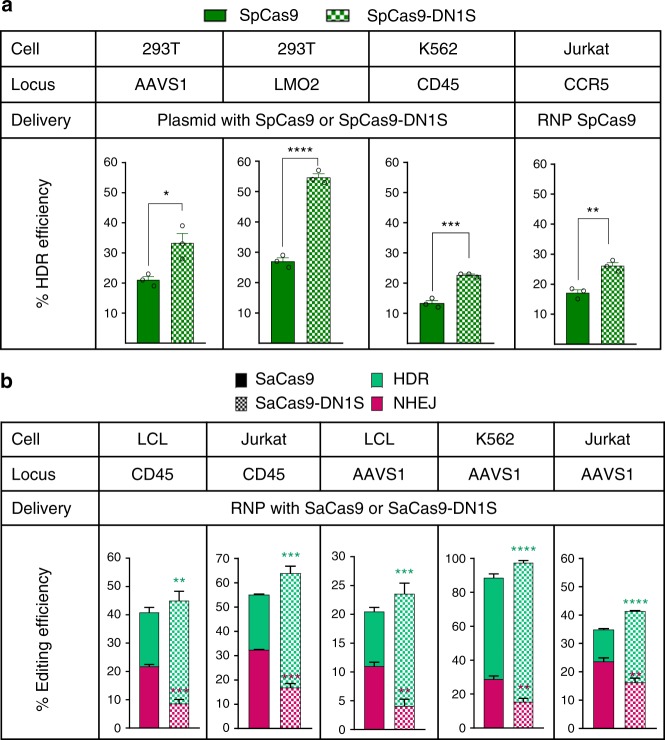
Cas9-DN1S stimulates HDR at different target genes in multiple cell lines. **a** Bar plots showing the HDR editing efficiency of SpCas9 or SpCas9-DN1S at the AAVS1 and LMO2 loci in 293T cells, the CD45 locus in K562 cells, and the CCR5 locus in Jurkat cells. SpCas9 or SpCas9-DN1S and the donor templates were delivered through the plasmid system in 293T and K562 cells. SpCas9 or SpCas9-DN1S were delivered by ribonucleoprotein (RNP) and the CCR5-GFP donor by rAAV6 in Jurkat cells. HDR efficiency was determined by the percentage of GFP+ cells. The data are presented as the mean ± SEM of three independent electroporations. Black circles indicate individual data points. Statistics: Unpaired *t* tests, one tailed. *P < 0.05, **p < 0.01, ***p < 0.001, and ****p < 0.0001. **b** Stacked bar plots showing the NHEJ and HDR editing efficiency of SaCas9 or SaCas9-DN1S at the CD45 and AAVS1 loci in LCL cells, at the AAVS1 locus in K562 cells, and at the CD45 and AAVS1 loci in Jurkat cells. SaCas9 or SaCas9-DN1S was delivered as RNP and donor templates were delivered via rAAV6 vectors. HDR efficiency was quantified by the percentage of GFP+ cells. NHEJ efficiency at the AAVS1 locus was determined by amplifying the edited locus by PCR and performing TIDE assay. When targeting the CD45 locus, NHEJ efficiency was determined by the CD45- population by flow cytometry, and confirmed by TIDE assay. The data are presented as the mean ± SEM of 3–5 independent electroporations. Statistics: Unpaired *t-*tests, one tailed comparing NHEJ (magenta asterisks) or HDR (green asterisks) separately: **p < 0.01, ***p < 0.001, and ****p < 0.0001. Source data is available in Source Data file

We then tested the ability of SpCas9-DN1S to precisely target a GFP reporter in 293T cells to two other gene loci: *AAVS1* and *LMO2* (Supplementary Table 1). The SpCas9-DN1S improved HDR efficiency (GFP+ cells) on average from 21% to 33.3% at the *AAVS1* locus, and from 27% to 54.6% at the *LMO2* locus (Fig. 3a). We also tested SpCas9-DN1S in three hematopoietic cell lines, K562 cells, EBV-immortalized normal B cells (LCL) and Jurkat T cells. Additionally, we targeted two additional gene loci that are not as accessible to HDR, albeit efficient at NHEJ repair: the *CD45* gene locus, a recently reported cell-surface gene editing reporter system^16,33^ and the *CCR5* locus (Supplementary Table 1)^34^. We targeted GFP downstream of the *CD45* gene promoter, or in the *CCR5* locus, to be able to detect HDR by flow cytometry^16^. Hence at the *CD45* locus, while NHEJ would result in loss of CD45 expression, HDR would result in GFP+ CD45+ cells. HDR at the *CCR5* locus also resulted in GFP+ cells. SpCas9-DN1S protein significantly increased HDR from 13% and 17% with SpCas9 to 23% and 26% with SpCas9-DN1S at the CD45 and CCR5 locus in K562 and Jurkat cells, respectively (Fig. 3a**;** Supplementary Fig. 10a); but with increasing the amounts of AAV donor template, we optimized HDR frequencies to approximately 60% and 70% with SpCas9 and SpCas9-DN1S, respectively (Supplementary Fig. 10b).

We also confirmed that the effect of DN1S in reducing NHEJ and improving HDR is observed in hematopoietic cells when it is fused to the SaCas9 nuclease. As expected, SaCas9-DN1S also significantly improved the HDR frequency in three different cell lines: K562 cells, LCL B cells and Jurkat T cells (Fig. 3b). In LCL, HDR increased from 9.5% to 19.5% at the *AAVS1* locus (Fig. 3b), and from 19% to 36% at the *CD45* locus. In K562 cells at the *AAVS1* locus, with SaCas9 alone, we were able to optimize HDR efficiencies to 60% with SaCas9, but ~30% of editing events were NHEJ-mediated knockout events. However, with SaCas9-DN1S, HDR frequencies reached levels of 78–86% HDR, with only 10–15% NHEJ repair at this locus (Fig. 3b). In Jurkat T cells, HDR increased from 11% to 25% at the *AAVS1* locus and from 22% to 47% at the CD45 locus with SaCas9 and SaCas9-DN1S, respectively (Fig. 3b). Like SpCas9-DN1S (Supplementary Fig. 9), SaCas9-DN1S also remarkably decreased NHEJ frequency in different cell lines and gene loci tested (Fig. 3b), while improving HDR.

By increasing the amount of AAV donor delivery, we investigated whether the high level of GFP+ cells were indeed reflective of HDR, since AAV can integrate in the absence of nuclease^35^. Off-target integration would very rarely result in GFP expression with SaCas9/CD45 gRNA or SaCas9-DN1S/CD45 gRNA, since GFP in the donor does not have its own promoter, and is designed to express GFP only if targeted downstream of the CD45 promoter in-frame with the *CD45* gene; this would result in dual expression of GFP and CD45, and NHEJ events result in CD45- cells^16^. Indeed, this was the case when high concentrations (high MOI) of *CD45*-targeted GFP donor in single-stranded AAV (ssAAV) or self-complementary AAV (scAAV) alone were provided as controls (Supplementary Fig. 11) with barely detectable GFP+ cells, while high levels of HDR (GFP + CD45 + cells) were observed with SaCas9-DN1S.

When the GFP donor in AAV is targeted to the *AAVS1* locus or the *LMO2* locus, or the CD18 donor is targeted to the *AAVS1* locus, GFP and CD18 are driven by their own promoters present in the donor template, and hence off-target random integration of AAV donor would result in GFP+ or CD18+ cells. It is to be noted that we analyzed reporter expression by flow cytometry at 12–14 days in culture as a routine, so that any GFP or CD18 expression is not from transiently present AAV virus in cells and is measured from cells that have undergone HDR. To determine the frequency of off-target integrations of the GFP donor in AAV in this scenario, we derived individual clones from K562 and Jurkat cells edited with SaCas9/AAVS1 gRNA and SaCas9-DN1S/AAVS1 gRNA with GFP-AAV donor template at high AAV MOI, and confirmed by PCR the presence of GFP and HDR (using primers upstream of the 5′ homology arm and within the promoter region in the donor template) (Supplementary Fig. 12). AAV donor alone had 0.2–2% random integration. The background off-target integration of AAV in the presence of Cas9 or Cas9-DN1S was slightly higher, likely because AAV tends to integrate more efficiently with increased DNA damage response in cells^36^ and nuclease induced DSBs activate the cellular DNA damage response machinery. Importantly, however, off-target AAV integration appeared lower with SaCas9-DN1S (2–4%) than SaCas9 (4–20%). We confirmed that HDR occurred as designed by sequencing the region spanning the 5′ and 3′ homology arms and found that the sequence aligned perfectly with the expected targeted sequences. Hence, the increased HDR seen with the SaCas9-DN1S fusion, as compared to SaCas9, was not from increased off-target integrations of the AAV donor, and if off-target integrations were present, they were similar, and indeed higher in the Cas9 edited cells.

Notably, the degree of HDR varied at different loci and in different cell types^37^, but overall, the Cas9-DN1S fusion nucleases increased HDR by 2–3 fold in all the cell lines and gene loci tested, with a 3–4 fold reduction in NHEJ. However, at one gene locus in one cell type, we observed a minimal increase in HDR frequency at the CD45 gene locus in K562 cells with SaCas9-DN1S, although at this locus, there was a significant reduction in the NHEJ frequency from 60% to 20%, and therefore, a significantly improved HDR to NHEJ ratio (Supplementary Fig. 13). It is to be noted, however, that SpCas9-DN1S did increase HDR at this locus (Fig. 3a). Notably, while the difference between the HDR with Cas9 and Cas9-DN1S narrowed at high HDR efficiencies (though was still statistically highly significant), there were more GFP^bright+^ cells in the Cas9-DN1S group within the GFP+ (HDR) population (see Supplementary Figure 12a), suggesting more bi-allelic HDR may be occurring with Cas9-DN1S.

The GFP donor template targeting the CD45 locus, provided either as single-stranded DNA (in single-strand AAV; ssAAV) or as double-strand DNA (in self-complementary AAV; scAAV) showed a comparable increase in HDR with SaCas9-DN1S as compared to SaCas9 (both SaCas9 and SaCas9-DN1S were provided as RNPs). RNPs peak in cells at 4–6 h and are degraded by 24 h^31^, a time-period insufficient for second-strand synthesis of ssAAV^38,39^ (Supplementary Fig. 11), showing that single-strand and double-strand DNA donors in AAV increased HDR with Cas9-DN1S comparably.

### CD18 insertion in LAD patient-derived B lymphocytes

**Fig. 4:**
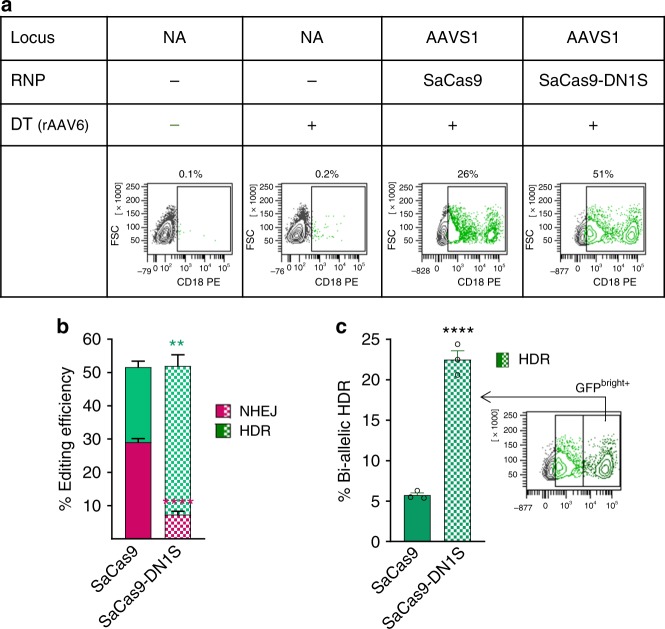
CD18 insertion at the AAVS1 locus in LAD patient-derived B lymphocytes. **a** Representative flow cytometry plots of EBV-immortalized LAD patient B-lymphocytes, electroporated for targeted integration of CD18 at the AAVS1 locus, showing fractions of HDR+ cells. Donor template (DT) was delivered via rAAV6. **b** Stacked bar plots showing quantification of HDR efficiency by flow cytometry and NHEJ efficiency by TIDE assay of SaCas9 or SaCas9-DN1S with AAVS1-CD18 rAAV6 donor template. The data are presented as the mean ± SEM of three independent electroporations. Statistics: unpaired t tests, one tailed comparing HDR (green asterisks) or NHEJ (magenta asterisks): **p < 0.01, ****p < 0.0001. **c** Relative frequencies of bi-allelic HDR detected by sorting the GFP^dim+^ and GFP^bright+^ cell populations as gated, based on the density and MFI of CD18 by flow cytometry. The sorted GFP^bright+^ population was confirmed to be bi-allelic HDR by PCR (shown in Supplementary Fig. 14). The data are presented as the mean ± SEM of three independent electroporations. Black circles indicate individual data points. Statistics: unpaired *t-*tests, one tailed: ****p < 0.0001. Source data is available in Source Data file

To test our fusion construct in a clinically relevant model, we attempted to genetically correct leukocyte adhesion deficiency (LAD) in patient-derived immortalized B-lymphocytes. LAD results in lack of cell-surface expression of the CD18 integrin β chain, resulting in defective adhesion of leukocytes for bacterial killing, and causing increased and chronic life-threatening infections^40^. In this disease, modest to low expression of CD18 via gene addition has been shown to not correct the disease phenotype in dogs with LAD^41^, while robust CD18 expression driven by vectors carrying strong viral promoters and enhancers has been shown to correct the disease^42^. We targeted the human CD18 cDNA, driven by the MND promoter, into the AAVS1 safe harbor locus by providing the donor template in an AAV6 vector along with SaCas9-DN1S protein and AAVS1 gRNA complex. We used equimolar amounts of SaCas9/gRNA or SaCas9-DN1S/gRNA to target CD18 into the AAVS1 locus. As expected, these LAD-derived B cells did not express CD18. Compared to SaCas9, the SaCas9-DN1S increased the CD18 expressing B cell population (cells that had undergone HDR) from 23% to 45%, and concomitantly, reduced error-prone NHEJ repair from 30% to 7% (Fig. 4a, b). When examined closely, we observed a large distinct CD18 bright cell population within the CD18 expressing HDR population in cells edited with SaCas9-DN1S (Fig. 4a). We sorted this CD18 bright population and found that these cells had undergone bi-allelic HDR as confirmed by PCR (Supplementary Fig. 14). Indeed, gating the CD18 bright population revealed that more than half the CD18+ HDR population had bi-allelic HDR with the fusion construct SaCas9-DN1S. Hence, SaCas9-DN1S not only increased the overall HDR by 2-fold, it also increased the bi-allelic HDR frequency by five-fold, as compared to SaCas9 (Fig. 4c). Hence, at the allelic level, nearly 70% alleles were repaired by HDR by the SaCas9-DN1S nuclease, with 7% alleles repaired by NHEJ. In contrast, 28% alleles were repaired by HDR and a similar number of alleles (29%) repaired by NHEJ by SaCas9. These levels of precise correction by the SaCas9-DN1S, with minimal error-prone repair are clinically translatable.

To assess the impact of CRISPR/Cas9-DN1S on on-target specificity, we performed off-target analysis by sequencing at the top four off-target sites (OTS) of SpCas9 AAVS1 gRNA^43^ (which is known to produce high off-target editing^44^), four predicted OTS of SaCas9 AAVS1 gRNA, and three predicted OTS of SaCas9 CD45 gRNA (Supplementary Fig. 15a, c, e). While these are limited analyses, we did observe similar or significantly decreased off-target cutting efficiency of both SpCas9-DN1S and SaCas9-DN1S, at least at these predicted loci (Supplementary Fig. 15b, d, f), suggesting that fusing DN1S with Cas9 does not increase its affinity to cut more frequently at known OTS.

## Discussion

Our goal was to improve gene editing by increasing HDR while decreasing NHEJ. However, global suppression of NHEJ is particularly toxic to hematopoietic cells. To overcome this difficulty, we have tethered DN1S dominant-negative 53BP1 to Cas9. An optimal DN 53BP1 fragment, DN1S, was generated by maintaining domains required to recruit it to DSBs, while being devoid of domains that interact with effectors of NHEJ. This enabled a non-functional DN protein to compete with and/or displace endogenous 53BP1. In agreement with Canny et al.^15^, who utilized an untethered inhibitor of 53BP1, our results suggest that HDR is improved by reducing 53BP1 binding to Cas9-induced DSBs. However, these contrast to results reported by Paulsen et al.^17^, where a murine minimal FFR dominant negative 53BP1 by itself did not improve HDR by CRISPR-Cas9 in HEK293 cells, suggesting that a murine fragment may not be as effective in promoting HDR in human cells, unless combined with an HDR protein (Rad52).

We show significant toxicity of untethered DN1S in hematopoietic cells. While we find that the toxicity of untethered DN1S in HeLa cells is relatively modest compared to its effect on hematopoietic cells, DN1S tethered to Cas9 is not toxic. It is conceivable that HeLa cells are not as dependent on NHEJ-repair as Jurkat or K562 cells. Hence, the strategy proposed by Canny et al.^15^ resulting in global inhibition of NHEJ, may be applicable in cell types not heavily dependent on NHEJ, where a short exposure to a 53BP1 inhibitor may not be toxic, and the delivery of the Cas9-DN1S fusion may not be possible via AAV for in vivo applications. In this context, it should be noted that Canny et al.^15^ did not test the toxicity of untethered 53BP1 inhibition. Paulsen et al.^17^ reported that untethered mouse dn53BP1 was not toxic, but it also did not alter NHEJ frequency in HEK-293 cells, whether present alone or in combination with Rad52.

Interestingly, while DN1S increased HDR at most, but not all, loci tested, it inhibited NHEJ at all loci. Thus, utilization of DN1S tethered to Cas9 is expected to have a broad effect on decreasing mutagenesis associated with CRISPR/Cas9-mediated gene editing. Finally, we demonstrate that fusion to DN1S can significantly increase the frequency of bi-allelic HDR mediated by Cas9, thereby potentially improving correction of genetic deficiencies by gene editing, especially in diseases where gene dosage is important for a therapeutic benefit.

The CRISPR-Cas9-DN1S system we have developed here can be more clinically relevant for (i) boosting HDR-based precise genome editing that is therapeutically desirable, and for (ii) safety by specifically reducing NHEJ events at Cas9 induced breaks. More importantly, this effect is achieved at several different gene loci, in multiple hematopoietic and non-hematopoietic cells, with different Cas9 nucleases and donor templates delivered as dsDNA (plasmids, scAAV), or ssDNA (ssAAV). (iii) Further, fusion of DN1S to Cas9 does not globally affect the cellular NHEJ pathway or increase off-target editing/integrations. Therefore, Cas9-DN1S does not cause an appreciable threat to genome stability. Indeed, the DN1S fusion can likely work with any site-specific nuclease and perform as designed.

## Methods

### Human cell culture

Human embryonic kidney (HEK) 293T, HeLa, and the human hematopoietic cell lines K562 and Jurkat, were obtained from the American Type Culture Collection (ATCC). EBV transformed B-lymphoblast cells (LCL) were generated by immortalizing primary B lymphocytes with EBV by the Diagnostic Immunology Laboratory, CCHMC. Leukocyte adhesion deficiency (LAD) patient-derived B lymphocytes cells were EBV immortalized and were obtained from Dr. Dennis Hickstein at the National Cancer Institute (NIH). HeLa EJ5-GFP cells with an integrated I-SceI GFP reporter cassette for NHEJ assessment were kindly provided by Dr. Younghoon Kee at the University of South Florida. HeLa cells, K562 cells, and 293T cells were cultured in DMEM (Corning) supplemented with 10% fetal bovine serum (FBS; VWR), 1% l-glutamine (MP Biomedicals) and 1% Penicillin-Streptomycin (Lonza). LAD patient-derived B lymphocytes, Jurkat, and LCL cells were cultured in RPMI-1640 (Sigma) supplemented with 20% FBS and 1% Penicillin-Streptomycin.

### Generation and testing of 53BP1 DN constructs

Dominant-negative (DN) lentiviral constructs were designed based on the NCBI Protein P53BP1, accession Q12888 and NCBI Nucleotide TP53BP1 transcript variant 3 mRNA, accession NM_005657.2. The four initial constructs included (DN1) amino acids 1231–1711, (DN2) amino acids 1231–1277 followed by a TGS linker and ending with amino acids 1480–1644, (DN3) amino acids 1480–1644, and (DN4) amino acids 1480–1711. Sequences for DN1 and DN2, HA-NLS signals, and stop codon sites were ordered from Genscript. Fragments were amplified from the Genscript constructs by PCR and assembled by overlap-extension PCR^45^. In short, Q5 polymerase (NEB) was used to individually amplify the four 53BP1 fragments, HA-NLS sequences for the 1231 and 1480 start positions, and the stop codon regions for the 1644 and 1711 positions (Supplementary Table 2). These sequences were assembled in a second round of PCR with Q5 polymerase (NEB) without primers to anneal the sequences for 10 cycles, followed by amplification with primers for 40 cycles. Annealed amplicons were isolated by gel extraction. Amplicons were digested with SpeI and BstBI (NEB) for insertion into lentiviral backbone SSIN.MND.MCS between XbaI and BstBI (NEB). Resulting final constructs were confirmed by restriction digest and sequencing.

### Production of lentivirus

Lentiviral constructs were produced by transfection of 293T cells with transgene, Gag/Pol (∆8.9-SO), VSVG envelope, and pRSV-REV plasmids in a 4:3:1:1 ratio with 1 mg/ml polyethylenimine (PEI, Polysciences). Cells were first plated in T-225 flasks at a density of 30 × 10^6^ cells/flask and incubated overnight to allow for adherence. The next day, media was replaced and cells were transfected. Viral supernatant was collected 60 h post-transfection. Viral supernatant was first filtered, then ultracentrifuged to pellet viral particles. Particles were purified over a sucrose gradient and the final pellet resuspended in StemSpan SFEM (Stemcell Technologies) supplemented with 2% FBS. Lentiviral aliquots were stored at −80 °C until use.

### Western blot

HeLa cells were transduced with lentiviral vectors expressing five different HA-tagged recombinant 53BP1 proteins: DN1, DN1S, DN2, DN3 and DN4. Cells were lysed in 50 mM Tris HCl, pH 7.4, 250 mM NaCl and 5 mM EDTA, supplemented with protease and phosphatase inhibitors, and lysates resolved on polyacrylamide gels and transferred to nitrocellulose membranes^46^. Primary antibodies used were to HA (1:1000; Covance 16B2) and actin (1:1000; Santa Cruz sc-8432). Uncropped western blot images are provided in the associated Source Data file.

### Immunofluorescence microscopy

HeLa cells were transduced with lentiviral vectors expressing DN1, DN1S, DN2, DN3, or DN4, or an empty vector control. The transduced cells were plated onto coverslips and were either irradiated (2 Gy) or not irradiated. The irradiated cells were fixed with 2% paraformaldehyde for 20 min at room temperature (RT), 2 h after irradiation. Coverslips were incubated in 0.2% Triton X-100 at RT for 3 min, and then incubated with primary antibody for 1 h at 37 °C in dilution buffer^46^. Dilution buffer consisted of 3% BSA, 0.05% Tween 20, and 0.04% sodium azide. All DN constructs were checked for formation of foci with HA antibody (1:200; Covance 16B2). For DN1 and DN1S immunofluorescence, antibody dilutions were as follows: Endogenous 53BP1 (1:200; Novus NB100–304); γH2AX (1:200; EMD Millipore 05–636); RIF1 (1:200; Novus NBP2-47303); mouse αBRCA1 (1:50; Santa Cruz sc-6954); Rad51 (1:200; Santa Cruz sc-8349); and HA (1:200; Covance 16B2). Coverslips were incubated in secondary anti-mouse or anti-rabbit FITC or Cy3-conjugated antibodies (1:200; Jackson Immunoresearch 715-545-150 and 711-165-152) for 30 min at 37 °C. The coverslips were then mounted onto glass slides with Vectashield mounting medium containing DAPI (Vector Laboratories). Images in Figs. 1 and 2a, and images in Supplementary Figures, were taken using a ×60 objective on a Nikon Eclipse Ti microscope with a Neo/Zyla camera driven by NIS Elements AR 4.50 Imaging software. The cells were counted to be positive based on ≥3 foci/nucleus. The data are presented as the mean ± SEM of three counts of 150 cells from three independent fields.

### EdU and BRCA1 immunofluorescence assay

HeLa cells were transduced with lentiviral constructs expressing DN1 or DN1S, or an empty vector control. The Click-iT EdU Alexa Fluor 488 Imaging Kit (Invitrogen) was used following the manufacturer’s recommendations for EdU staining. In short, the cells were labeled with 10 μM EdU for 2 h in culture. Cells were then fixed with paraformaldehyde, permeabilized, and washed once with PBS. Each coverslip was incubated with 50 μl of freshly prepared Click-iT reaction cocktail for 30 min at RT. Coverslips were washed once for 5 min with PBS plus 3% BSA, and again for 5 min with PBS only. Rabbit anti-BRCA1 antibody (1:200; Cell Signaling 9010) was added to coverslips and incubated for 1 h, then washed three times for 5 min in PBS. Coverslips were incubated with donkey anti-rabbit Cy3 secondary antibody (1:200; Jackson ImmunoResearch 711-165-152), then washed with PBS and mounted onto glass slides with Vectashield mounting medium containing DAPI (Vector). The cells were counted based on EdU staining and the presence of ≥3 BRCA1 foci/nucleus. The data are presented as mean ± SEM of three counts of 150 or more cells from three independent fields.

### Generation and testing of Cas9-DN fusion constructs

pX458M, a kind gift from Dr. Yueh-Chiang Hu of the Transgenic Animal and Genome Editing Core at CCHMC, was used to generate Cas9 dominant-negative fusions. Overlap-extension PCR^45^ was used to generate the dominant negative fusions (DN1, DN1S, DN2, and DN2L) at the C-terminus of Cas9, preceded by an NLS and 2A sequence (primer sequences in Supplementary Table 3). The overlap-extension PCR amplicons were inserted between SanDI and FseI of the pX458M construct, resulting in constructs containing the U6-gRNA cassette, CBH promoter, 3xFLAG-tagged SpCas9 fused to the dominant negative 53BP1, and a 2A-eGFP reporter. For use with the traffic-light reporter (TLR) reporter system, the eGFP was replaced with eBFP2, originating from pX330-BFP (Addgene 64323), between FseI and BsrGI. For insertion of gRNA, the BbsI site behind the U6 promoter was digested to allow for ligation of gRNA-specific annealed oligos containing BbsI overhangs. For the TLR system, Rosa26-1 gRNA was inserted at the BbsI site (Supplementary Table 1). Additional gRNAs for the following targets were also inserted into the SpCas9-DN1S and SpCas9 control constructs: LMO2, AAVS1, and CD45 (Supplementary Table 1).

For colony forming assay and immunofluorescence microscopy, Cas9-containing self-inactivating (SIN) lentiviral transgene constructs were generated to contain BsmBI sites for insertion of the U6-gRNA cassette, followed by an EFS promoter, HA-tagged SpCas9-2A-mCherry, and optimized PRE. To insert the 53BP1 DN1S fusion, the DN1S fragment was generated by PCR to introduce a SacII site on the 3′ end (Supplementary Table 3). The DN1S fragment was inserted between PmlI in the Cas9 and SacII ahead of the 2A signal. To insert gRNA, the construct was digested with BsmBI and the U6-gRNA cassette from the pX458M plasmid was removed between AflIII and Acc65I (NEB). The overhangs generated by the BsmBI sites correspond to the AflIII and Acc65I overhangs, allowing for ligation of the U6-gRNA cassette ahead of the EFS promoter. For these constructs, the human CD45 gRNA was inserted. Lentivirus was produced as described above.

Alternate truncated NGFR (tNGFR) vectors expressing DN1S or Cas9-DN1S were also developed. For the tNGFR DN1S construct, DN1S was moved from the SSIN.MND.DN1S lentiviral construct into a lentiviral backbone containing a separate cassette for CMV.tNGFR between XbaI and SalI (NEB). For the tNGFR Cas9-DN1S construct, SpCas9.TGS.DN1S was moved from the PX458M plasmid construct into the CMV.tNGFR lentiviral construct between NcoI and MfeI (NEB). Both DN1S and Cas9-DN1S tNGFR constructs were driven by the MND promoter.

Dead Cas9 D10A and N863A mutations were generated with the Agilent QuikChange XL II kit using a Cas9-containing plasmid as a template (primers listed in Supplementary Table 3). Clones were screened for the correct sequence, then used to generate dSpCas9 and dSpCas9-DN1S constructs without gRNA and with gRNA to target the human CD45 locus.

### Western blot and immunofluorescence of Cas9-DN constructs

HeLa cells were transfected with plasmids expressing GFP reporter along with FLAG-tagged fusion proteins: SpCas9-DN1, SpCas9-DN1S, SpCas9-DN2 and SpCas9-DN2L. The transfected cells were sorted for GFP+ cells and lysed for western blotting as described above. Primary antibodies used were to FLAG (1:1000; Sigma F1804) and actin (1:1000; Santa Cruz sc-8432). To assess the stability of Cas9-DN1S with and without gRNA, HeLa cells were transduced with lentiviral vectors expressing HA-tagged dCas9-DN1S or dCas9-DN1S/CD45 gRNA. Cells were collected and lysed as described earlier. Primary antibodies used were to HA (1:1000; Covance 16B2) and actin (1:1000; Santa Cruz sc-8432).

For immunofluorescence microscopy, cells were transfected with lentiviral vectors expressing HA-tagged DN1S, dCas9, dCas9-DN1S, and dCas9-DN1S/CD45 gRNA. Cells were plated on coverslips, fixed, and stained as described earlier. Antibody dilutions were as follows: Endogenous 53BP1 (1:200; Novus NB100-304); γH2AX (1:200; EMD Millipore 05-636); RIF1 (1:200; Novus NBP2-47303); rabbit αBRCA1 (1:200; Cell Signaling 9010); Rad51 (1:200; Santa Cruz sc-8349); and HA (1:200; Covance 16B2).

### NHEJ assay in HeLa cells

HeLa EJ5-GFP cells containing a promoter separated from a GFP coding region by a puromycin gene flanked by two I-SceI recognition sequences were used to measure disruption of NHEJ by DN1S constructs^30^. HeLa EJ5-GFP cells were transduced with lentiviral vectors expressing DN1S or SpCas9-DN1S. After stable integration, transduced cells were plated at a density of 30,000 cells per well in triplicate in a 48-well plate and incubated overnight prior to transfection. Cells were transfected by Lipofectamine 3000 (Thermo Fisher Scientific) with 500 ng of I-SceI plasmid (Addgene, 26477). Readout was performed by flow cytometry for GFP+ cells 72 h after transfection on a BD FACSCanto instrument.

### dCas9-DN1S co-localization with centromeres

A gRNA for targeting centromeres was designed to target the 16 bp conserved CENPB box^29^ (Supplementary Table 1). Analysis with NCBI BLAST indicated that the 16 bp sequence occurs at over 93,000 genomic locations. The chosen 23 bp gRNA plus PAM sequence was found to occur at over 26,000 genomic locations. A U6-CENPB gRNA cassette was ordered from IDT and inserted into dSpCas9 and dSpCas9-DN1S mCherry-expressing lentiviral vectors between XbaI and SalI (NEB) upstream of the EFS promoter. HeLa cells were transduced with dSpCas9/CENPB gRNA, dSpCas9-DN1S/CENPB gRNA, or dSpCas9-DN1S/CD45 gRNA constructs and sorted by a BD FACSAria II instrument for mCherry+ cells. Cells were plated on coverslips, fixed, and stained as described above. The following antibodies were used for immunofluorescence microscopy: CENPB antibody (1:200, Active Motif 61288) and HA (1:200, Covance 16B2). Images were collected using a Nikon C2 Confocal microscope driven by NIS Elements Imaging software, version 4.30.

### Colony forming assay

HeLa cells were transduced with five different lentiviral vectors, scrambled shRNA, 53BP1 shRNA (TRCN0000018866, Sigma), Cas9-gRNA, Cas9-DN1S-gRNA, dCas9-DN1S-gRNA and DN1S, or treated with 10 µM NU7441 (Selleckchem) for 20 h. Cells transduced with empty lentiviral vectors were used as controls. Cells transduced with 53BP1 shRNA were selected in puromycin (final concentration: 5 µg/ml). Cells transduced with Cas9-gRNA, Cas9-DN1S-gRNA, dCas9-DN1S-gRNA and DN1S lentivirus vectors, all encoding the mCherry fluorochrome, were sorted for mCherry positive cells. NU7441 treated cells, puromycin selected cells, and mCherry sorted transduced cells were subjected to gamma irradiation (IR) at different doses: 0.25 Gy, 0.5 Gy, 1 Gy, 2.5 Gy and 5 Gy. IR was delivered with a J L Shepherd Mark I Model 68 A Cesium 137 irradiator. 100–200 treated cells were plated in 6 well plates, and allowed to grow. Cells that retained viability despite IR formed colonies, and after 10–14 days, colonies were stained with Crystal Violet Solution (Sigma) following the manufacturer’s instructions and counted.

### Traffic light reporter assay by flow cytometry

Traffic light reporter (TLR) constructs were ordered from Addgene (64323, 64322, 64216, and 64215) and transfected into 293T cells, as described to target the TLR reporter into the AAVS1 locus to generate a TLR 293T reporter cells ^5^. The following eBFP2-containing constructs were transfected into TLR 293T cells by Lipofectamine 3000 (Thermo Fisher Scientific): SpCas9-DN1, SpCas9-DN1S, SpCas9-DN2, SpCas9-DN2S, and SpCas9 control with the pTLR Repair Vector donor template. Transfected TLR 293T cells were checked for the percentage of cells positive for BFP at day 2 to determine transfection efficiency of Cas9 constructs. At day 15, transfected TLR 293T cells were analyzed for the percentage of cells that were positive for RFP and Venus on a LSR-II flow cytometer (BD) to check NHEJ and HDR efficiency, respectively.

### Additional fusion constructs tested in TLR 293 T cells

To further optimize the Cas9-fusion 53BP1 construct, the orientation of the DN1S and the linker sequence fusing DN1S to SpCas9 were modified. We compared the N-terminal and C-terminal fusions of Cas9 to DN1S with the TGS linker (ACAGGGTCCACAGGATCCACAGGCAGCACAGGGAGCATGGGA) and the N-terminal fusion with the XTEN linker^47^ (AGCGGCAGCGAAACCCCCGGCACTAGT GAGTCCGCCACCCCCGAAAGT). Geneblocks were ordered from IDT to generate these replacements.

### HDR with plasmid constructs

For HDR at *AAVS1* and *LMO2*, 293T cells were transfected with Lipofectamine 3000 (Thermo Fisher Scientific) and the appropriate SpCas9 and SpCas9-DN1S gRNA-containing plasmids, and donor template plasmids with homology arms for *LMO2* or *AAVS1* with a self-promoted GFP cassette. Transfection efficiency was assessed by BD FACS LSR II at 72 h post-transfection. At 15 days post-transfection, GFP+ events were recorded by BD FACS LSR II to determine the percentage of HDR events. %HDR reported was normalized to the transfection efficiency, or percentage of donor and Cas9 double positive cells collected at 72 h post-transfection.

For HDR at the *CD45* locus, K562 cells were electroporated with SpCas9 or SpCas9-DN1S plasmids containing the CD45 gRNA plus a CD45-GFP plasmid donor using the Neon^TM^ Transfection System (Thermo Fisher Scientific). Cells were sorted for the double positive Cas9 and donor population 48 h after electroporation. Later, the cells were checked for GFP and CD45 (PE-Cy7, BD Biosciences 557748) expression by a BD LSR II instrument. HDR was determined by the %GFP+ CD45+ population.

### Production of fusion Cas9 proteins

To generate a plasmid capable of producing SpCas9-DN1S protein, pMJ806 (Addgene, 39312) was modified to include the TGS-53BP1 fusion. pMJ806 was digested between two BamHI sites at the 3′ end of the Cas9 sequence. Two Geneblocks (IDT), one containing just the 3′ end of the SpCas9 plus a 2XNLS sequence, and another containing the 3′ end of SpCas9 plus the TGS linker, DN1S, and a 2XNLS sequence, were ordered from IDT. These Geneblocks were digested with BamHI and ligated into the pMJ806 backbone. Additionally, a HA tag was added to the N-terminus of the SpCas9 protein by replacing the 5′ end of the SpCas9 sequence of pMJ806 between SpeI and SwaI. Colonies were screened by restriction digest to confirm orientation of the insert. Once a positive clone was isolated, the construct was confirmed by sequencing. The resulting plasmids, HA-SpCas9-NLS and HA-SpCas9-DN1S-NLS, were sent to QB3 Macro Lab at UC Berkeley for protein production. HA-SpCas9-DN1S-NLS was later modified to replace SpCas9 with SaCas9.

### RNP assembly

SpCas9 gRNA sequences for *LMO2* and SaCas9 gRNA sequences for *AAVS1* and *CD45* (Supplementary Table 1) were identified using the Benchling software. SpCas9 *AAVS1*, *CD45*, and *CCR5* gRNA sequences (Supplementary Table 1) were chosen from previous publications^16,34,43^. SpCas9 gRNAs were synthesized using the GeneArt Precision gRNA Synthesis Kit (Thermo Fisher Scientific). SaCas9 gRNAs were synthesized using a double-strand DNA template (ordered as a Geneblock from IDT) containing the T7 promoter, the guide sequence, and the SaCas9 TRACR sequence. The gRNA was synthesized using the HighScribe T7 High Yield RNA Synthesis (NEB) kit following the short RNA synthesis protocol. The resulting sgRNAs were purified with the MEGAclear Transcription Clean-Up Kit (Thermo Fisher Scientific). The concentration of the synthesized sgRNA was determined using a Nanodrop spectrophotometer (Life Technologies). To use in LAD patient-derived B lymphocytes, the synthetic chemically-modified single gRNA (sgRNA) for SaCas9 targeting the AAVS1 gene locus was purchased from Synthego. Lyophilized sgRNA was resuspended in nuclease-free 1X TE buffer (10 mM Tris HCL, 1 mM EDTA, pH 8.0) for a final concentration of 30 µM. The recombinant proteins: SpCas9-NLS, HA-SpCas9-NLS, HA-SpCas9-DN1S-NLS, SaCas9-NLS or HA-SaCas9-DN1S-NLS were produced by QB3 Macro Lab, UC Berkeley. Recombinant Cas9 or the Cas9-DN1S variant were stored at 40 µM in 20 mM HEPES-KOH, pH 7.5, 150 mM KCl, 10% glycerol and 1 mM DTT. RNP complexes (60 pmol of sgRNA and 40 pmol Cas9 or Cas9-DN1S nuclease) were assembled by incubating at room temperature for 15–30 min. RNPs were electroporated immediately after complexing.

### Delivery of RNP gene editing components

Immediately before electroporation, cells were centrifuged for 5 min at 100 × *g*, aspirated, and resuspended in 5 µl Neon electroporation Buffer R and 5 µl Cas9 RNP complex per 150,000–200,000 cells. The resuspended cells were electroporated using the Neon^TM^ Transfection System 10 µl kit (ThermoFisher) with the following electroporation parameters: 1350 V/30 ms/1 pulse for LAD patient-derived B lymphocytes and LCL cells, 1450 V/10 ms/3 pulses for K562 cells, and 1325 V/10 ms/3 pulses for Jurkat cells. The rAAV6 donors were added 15 min post-electroporation.

### Viability assay

To assess the viability of Cas9 and Cas9-DN1S, or the combination of DN1S and Cas9, K562 and Jurkat cells were first transduced with DN1S-expressing lentivirus or empty vector mock lentivirus. K562 cells with DN1S were electroporated with SaCas9 RNP with AAVS1 gRNA and AAVS1-GFP donor AAV, and K562 cells with empty vector were electroporated with either SaCas9 RNP or SaCas9-DN1S RNP with AAVS1 gRNA and AAVS1-GFP donor AAV. Jurkat cells with DN1S were electroporated with SaCas9 RNP with CD45 gRNA and CD45-GFP donor AAV, and Jurkat cells with empty vector were electroporated with either SaCas9 RNP or SaCas9-DN1S RNP with CD45 gRNA and CD45-GFP donor AAV. Cells were stained with eFluor^TM^ 780 fixable viability dye (eBioscience) and assessed by flow cytometry 24, 48, and 72 h post-electroporation. Cells that remained in culture for 14 days were assessed for HDR by flow cytometry for GFP+ cells in the K562 AAVS1 experiment, or GFP+ CD45+ cells in the Jurkat CD45 experiment (CD45 PE-Cy7 antibody: BD Biosciences 557748).

### AAV vectors and production

The rAAV6 donor for CD45-GFP HDR was generated by replacing the CB-GFP cassette of dsAAV-CB-GFP with a cassette including two 650 bp homology arms and a GFP-2A sequence linked to the start codon of the *CD45* CDS. To generate a single-stranded AAV donor, the CD45-GFP cassette and homology arms were cloned into the pAV backbone between EagI (NEB) restriction sites. All constructs were checked by restriction digest and sequencing for correctness.

The pAV-AAVS1-MND-CD18-pA donor construct was generated by inserting the CD18 cDNA between *AAVS1* homology arms. The *AAVS1* homology construct was synthesized by Genscript in a pUC57 backbone, with both homology arms spanning 550 bp on either side of the gRNA target sequence. A multiple cloning site was inserted within the gRNA target sequence to prevent recognition of the donor by the Cas9-gRNA complex. An MND-CD18-BGH polyA cassette was inserted between the homology arms at the multiple cloning site. Once the plasmid construct was verified, the complete AAVS1-CD18 cassette was removed between SrfI and NruI (NEB) and inserted into the pAV backbone between PmlI and BamHI (NEB) by first blunting any overhangs with Klenow Large Fragment followed by T4 ligation (NEB). Constructs were checked for orientation and sequence correctness. A GFP version of pAV-AAVS1-MND-CD18-pA was generated by replacing MND-CD18 with MND-eGFP between XbaI and NotI (NEB).

The CCR5 donor was generated by replacing the *AAVS1* homology arms of the pAV-AAVS1-MND-GFP-pA cassette with 600 bp homology arms for *CCR5* between EagI and XhoI (NEB) on the 5′ end and EcoNI and EagI (NEB) on the 3′ end. The *CCR5* homology sequences were ordered as Geneblocks from IDT.

Production of rAAV has been previously described^48–50^. In summary, 293T cells were expanded in complete media containing DMEM, 10% FBS, 1% L-glutamine, and 1% Penicillin/Streptomycin. The day before transfection, 12–14 × 10^6^ cells were plated in 15 cm dishes with 20 mL of complete media. The AAV2 transgene, AdHelper, and AAV2 Rep/AAV6 Cap plasmids were combined with serum-free DMEM and polyethylenimine (PEI, Polysciences) and incubated at room temperature for 10 min. Media was changed immediately before adding the transfection mixture. Transfected cells were cultured for 72 h at 37 °C, 5% CO_2_.

Cells were harvested by first collecting the media in 50 ml conical tubes, then by scraping the plates and washing with 0.5 M EDTA to remove all residual cells. The collected cells and supernatant were spun at 3000×*g* for 30 min. The cell pellets were resuspended with 15 ml of lysis buffer (150 mM NaCl, 50 mM Tris HCl, pH 8.5) and stored at −80 °C.

The cell pellets were thawed at 37 °C and snap frozen on dry ice and ethanol three times to ensure complete cell lysis. The cell lysate  was treated with 50 U/ml benzonase supplemented with 0.01 M MgCl_2_ and 0.5% sodium deoxycholate. The cell lysate was incubated at 37 °C for 1 h. After incubation, the cell lysate was centrifuged at 3000xg for 30 min. The supernatant was retained and the remaining cell debris discarded.

The cell lysate was prepared for purification using the iodixanol gradient method. Optiprep Density Gradient Medium iodixanol (Sigma) was diluted to 15%, 25%, 40%, and 54% with 1X PBS supplemented with 1 mM MgCl_2_ and 2.5 mM KCl. The 15% layer was additionally supplemented with 1 M NaCl. A Quick-Seal tube (Beckman Coulter) was first loaded with 14 ml of sample, then underlaid with 9 ml of 15%, 6 ml of 25%, 5 ml of 40%, and 5 ml of 54%. The tube was topped off with sample or 1X PBS to prevent tube collapse during ultracentrifugation. In brief, samples were ultracentrifuged with the Beckman Coulter 70Ti rotor at 170,000xg for 2 h using slow acceleration and deceleration. The AAV-containing fraction was recovered from the ultracentrifuge tubes by collecting the 40/54% iodixanol interphase with a needle and syringe. The AAV was transferred to a 10 K MWCO Slide-a-Lyzer G2 (Thermo Fisher Scientific) dialysis cassette and stirred in 1X PBS (HyClone) in a cold room overnight. The 1X PBS was replaced with fresh 1X PBS in the morning and allowed to stir an additional 2 hr. After 2 h, the 1X PBS was replaced with 1X PBS containing 0.5% d-Sorbitol (Sigma) and stirred for another 2 hr. After dialysis, the AAV-containing fraction was removed from the cassettes and concentrated in a 15-ml Amicon Ultra concentrator tube (Millipore) down to 500–1000 µL volume. The retentate was aliquoted in siliconized Eppendorf tubes and stored at −80 °C for future use.

### HDR by flow cytometry

K562, LCL, and Jurkat cells receiving RNP and AAVS1-GFP or CCR5-GFP rAAV6 donor were checked 10–15 days post-electroporation for GFP + cells using a BD FACSCanto. K562, LCL, and Jurkat cells receiving RNP and CD45-GFP rAAV6 donor were labeled with CD45 anti-human PECy7 antibody (BD Biosciences 557748). Cells were analyzed by a BD LSR II and those that were CD45+ and GFP+ were considered positive for HDR. NHEJ was determined by the CD45- population and confirmed by TIDE assay. LAD patient-derived B lymphocytes edited with AAVS1-CD18 donor were labeled with anti-human PE antibody for CD18 (BioLegend 302108) prior to analysis of the percentage of cells positive for CD18. NHEJ at the *AAVS1* locus was determined by TIDE assay.

### Genomic DNA isolation and TIDE assay

Transfected (edited) cells were cultured and harvested at days 7–12. Cell pellets were lysed with Cell Lysis Solution (Qiagen) and protein contamination removed with Protein Precipitation Solution (Qiagen). Genomic DNA (gDNA) was pelleted with isopropanol, washed twice with 70% ethanol, air dried, and resuspended in nuclease-free H_2_O. All gDNA samples were quantified by Nanodrop. For assessing NHEJ, the region around the Cas9 cut site was amplified by PCR using EconoTaq PLUS GREEN 2X Master Mix (Lucigen) or KAPA HiFi Hot Start PCR Kit (Roche) with specific primers, as stated in Supplementary Table 4. PCR products were run on 1–2% agarose gels, gel purified with the NucleoSpin Gel and PCR Clean-up Kit (Macherey-Nagel) and subjected to Sanger sequencing at the CCHMC DNA Sequencing and Genotyping Core. The sequencing results were analyzed with TIDE software (https://tide-calculator.nki.nl)^51^. Percentages of sequences with indels were plotted.

Off-target sites for analysis of SpCas9 and SpCas9-DN1S at *AAVS1* were selected from previously published off-target sites^44^. Off-target sites for analysis of *AAVS1* and *CD45* gRNA off-target effects for SaCas9 and SaCas9-DN1S were selected using the off-target analysis tool in Benchling.

### qPCR for random integration

K562 and Jurkat cells edited with SaCas9 or SaCas9-DN1S, AAVS1 gRNA, and AAVS1-GFP donor were single-cell sorted for GFP+ cells into a 96-well plate with a BD FACSAria II cell sorter. Bulk sorted GFP+ cells were also collected and used as plate controls for this assay. Clones were allowed to expand in culture for 2 weeks prior to cell lysis for genomic DNA with QuickExtract DNA Extraction Solution (Epicentre). Clones were first assessed for DNA quality and GFP integration by multiplex qPCR for ApoB by HEX (IDT) and GFP by FAM (IDT) using iTaq Universal Probes 2X Supermix (Bio-Rad) on a Bio-Rad CFX96 Touch. Clones were next assessed for correct integration across the 5′ *AAVS1* homology region and into the MND promoter of the integrated donor template using iTaq Universal SYBR 2X Supermix (Bio-Rad) with melt curve analysis on a Bio-Rad CFX96 Touch. Specific PCR primers and program conditions are listed in Supplementary Table 5. Clones that did not have amplification of ApoB or GFP or had minimal amplification of ApoB with Ct > 35 were not considered. Clones were marked positive for correct integration if they had sufficient amplification of ApoB, GFP, and 5′ HDR, plus a melting temperature within ± 0.5 degrees of the plate controls. Clones that did not have sufficient amplification of 5′ HDR or did not have appropriate melting temperatures were marked as random integration events. Total correct integration and random integration events for each plate were represented in table format.

### PCR for Bi-allelic HDR at AAVS1

LAD patient-derived B lymphocytes were electroporated with SaCas9 RNP targeted to the *AAVS1* locus and provided AAVS1-CD18 donor in rAAV6. Cells were labeled with PE-CD18 antibody and sorted by FACS on a BD FACSAria II instrument for the CD18^bright^ population, after which DNA from the resulting population was extracted using the method described above. Multiplex PCR to amplify either wild-type or NHEJ only events and HDR events was performed with the KAPA HiFi Hot Start PCR Kit (Roche) using the primers and conditions listed in Supplementary Table 6. The AAVS1-WT-RV primer aligns outside of the region of the homology arm and the AAVS1-HR-FW3 primer aligns in the CD18 cDNA.

### Reporting summary

Further information on research design is available in the Nature Research Reporting Summary linked to this article.

## Supplementary information

Supplementary Information

Reporting Summary

Source Data

## Data Availability

The data presented in Figs. 1b, 1d, 1f, 1h, 2b–c, 2e, 3a–b, and 4b–c and Supplementary Figs. 1a, 2b–d, 3b, 4b, 5c, 6a–c, 7b–c, 8a–b, 9a–b, 10b, 11a–b, 12a–b, 13a, 14a, 15b, 15d, and 15f are provided as a source data file. All relevant data not provided in the source data file and associated materials can be made available by the authors upon reasonable request.
