# Supplementary material for: CRISPR-Cas9 fusion to dominant-negative 53BP1 enhances HDR and inhibits NHEJ specifically at Cas9 target sites
Source: Nat Commun. 2019 Jun 28;10:2866. doi: 10.1038/s41467-019-10735-7 (PMC6598984; doi:10.1038/s41467-019-10735-7)
Supplement: Supplementary file 1 — Supplementary Information [file 41467_2019_10735_MOESM1_ESM.pdf]

Supplementary Information

Jayavaradhan et al.

Supplementary Figures

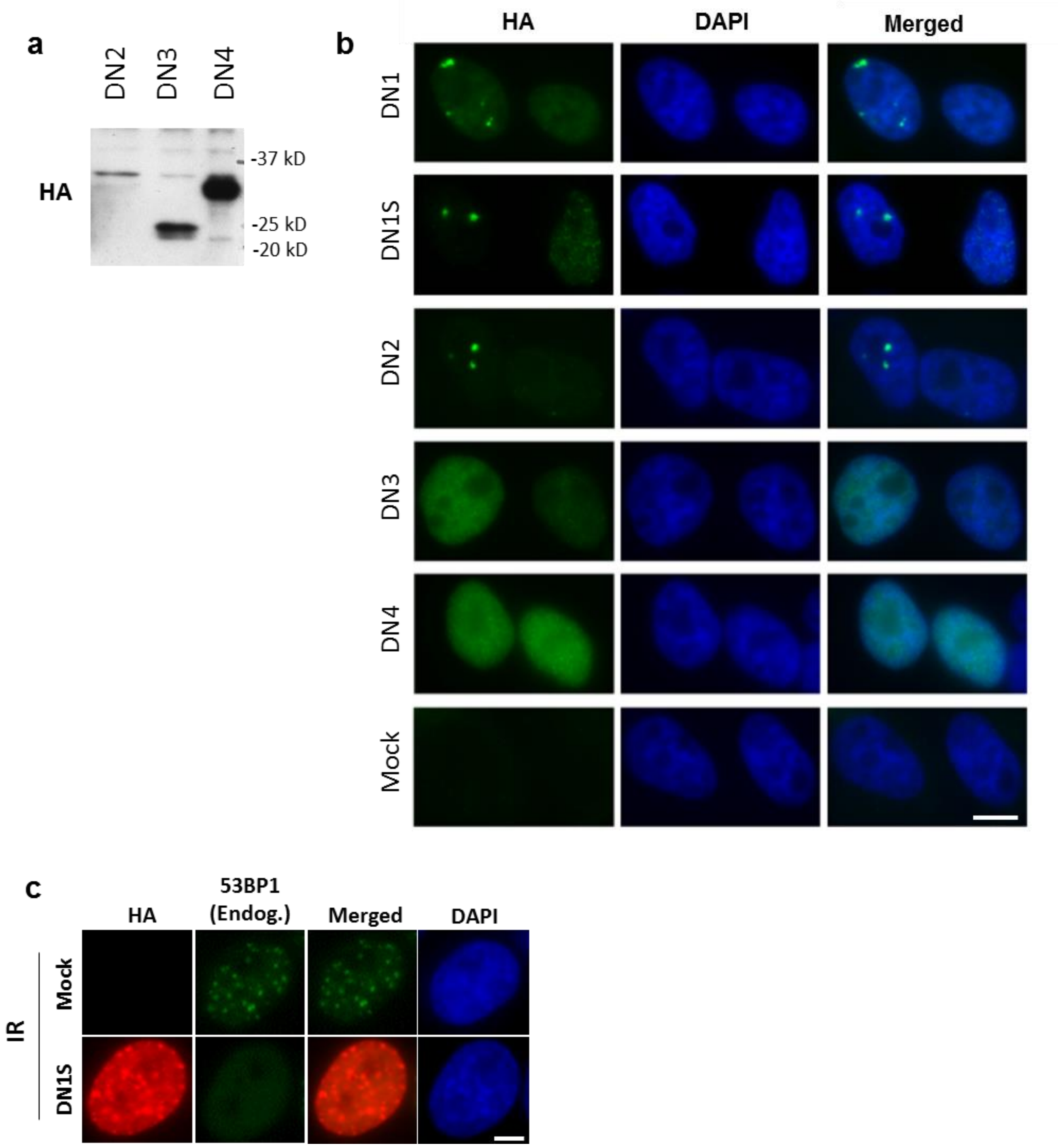

Supplementary Figure 1. Ability of DN 53BP1 fragments to form nuclear foci and DN1S to compete with endogenous 53BP1. **a**. Western blot showing the relative expression levels of DN2, DN3 and DN4, also shown in Figure 1b, at a higher exposure to better show DN2 and DN3. **b**. Representative immunofluorescence (IF) images showing foci forming ability of HA-tagged DN1, DN1S, DN2, DN3, DN4 and mock, stained for HA and DAPI. **c**. Representative IF images showing HA-tagged DN1S and endogenous 53BP1 recruitment to irradiation (IR)-induced DNA repair foci. The mock has no HA signal, whereas HA displaces all the endogenous 53BP1 foci at high levels of DN1S expression. Cells were exposed to 2 Gy IR and fixed 2 hours later. For panels b and c, scale bars represent 10μm. Source data is available in Source Data file.

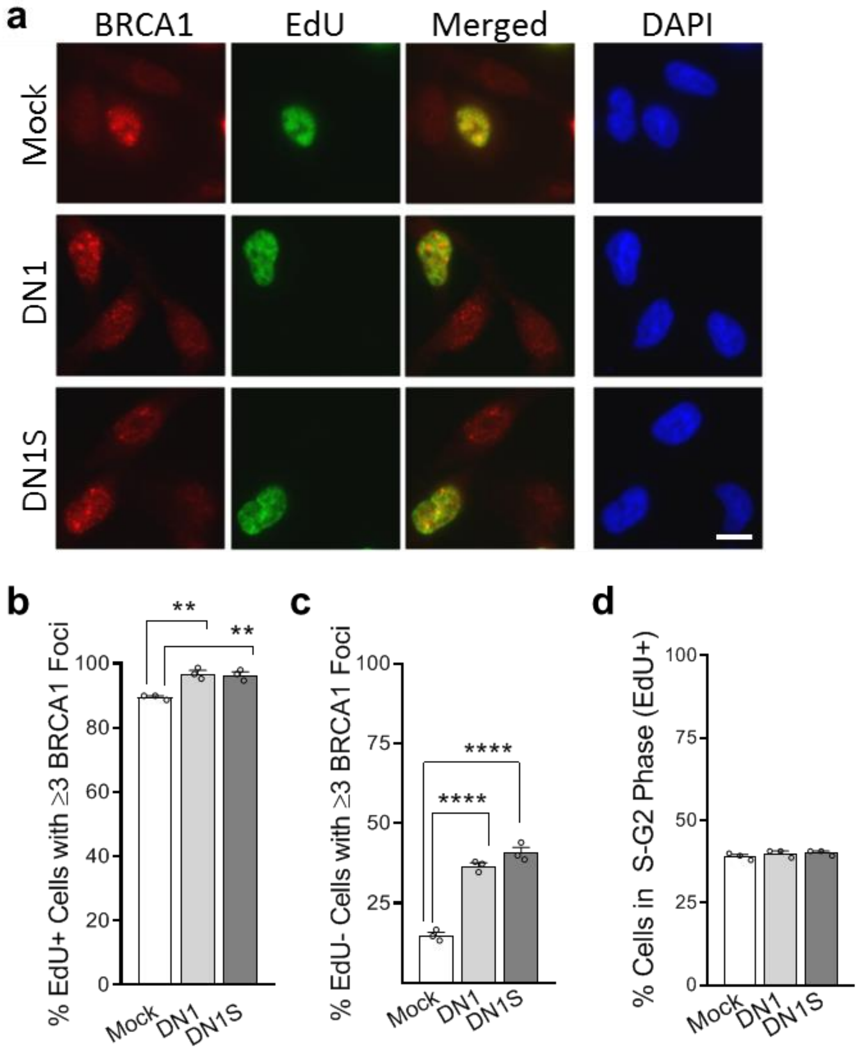

Supplementary Figure 2. DN1 and DN1S increase BRCA1 foci with a predominant increase in cells in G1 phase of cell cycle. a. Representative images displaying the presence or absence of BRCA1 foci in EdU-positive (EdU+) or EdU-negative (EdU-) HeLa cells expressing DN1 or DN1S, or which were mock transduced. Scale bar represents 20 μm. **b**. The percent of EdU+ (S and G2) cells, and **c.** EdU- (G0/G1) cells that display BRCA1 foci. **d.** The percent of HeLa cells transduced with DN1 or DN1S, or mock transduced populations that are positive for EdU is shown. Cells were labeled with EdU for 2 hours. Statistics for panels b-d: ANOVA. Each value represents the mean of three counts of 150 or more cells each from three independent fields ± SEM. Black circles indicate individual counts. Only significant data comparisons are marked: ** indicates $p<0.01$ and **** indicates $p<0.0001$. Source data is available in Source Data file.

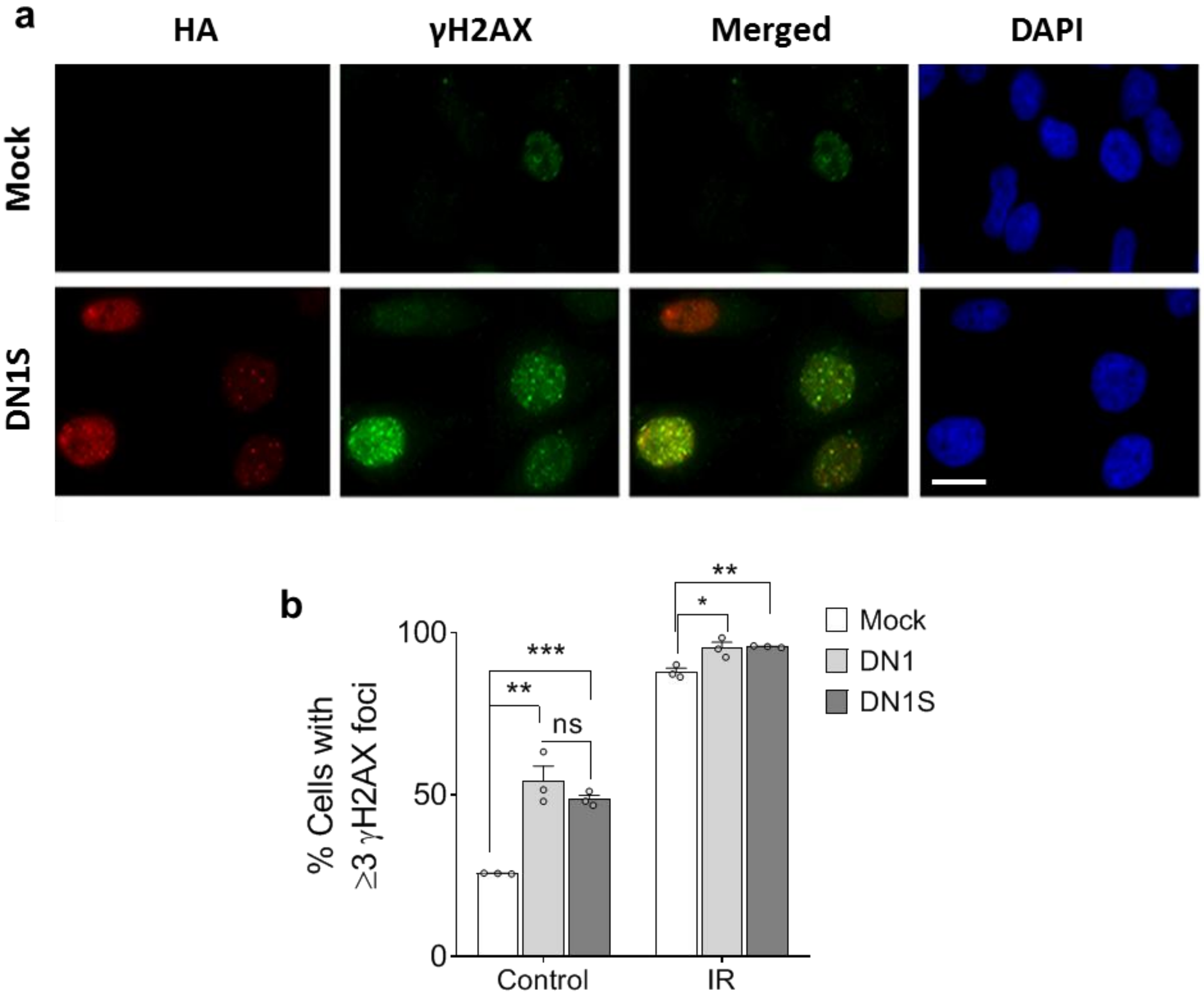

Supplementary Figure 3. DN1S increases γH2AX foci as compared to mock transfected cells. **a**. Representative immunofluorescence (IF) images showing HA-tagged DN1S and γH2AX recruitment to DNA repair foci. Scale bar represents 20 μm. **b**. Quantification of the number of cells with ≥3 γH2AX foci in control (unirradiated) cells or irradiated cells in the presence of DN1/DN1S or without (mock). Cells were fixed 2 hours after exposure to 2 Gy IR or were left unirradiated (control). The data are presented as the mean ± SEM of three counts of 150 cells each from three independent fields. Black circles indicate individual counts. Statistics: ANOVA. ns indicates not significant, * indicates p<0.05, ** indicates P<0.01 and *** indicates p<0.001. Source data is available in Source Data file.

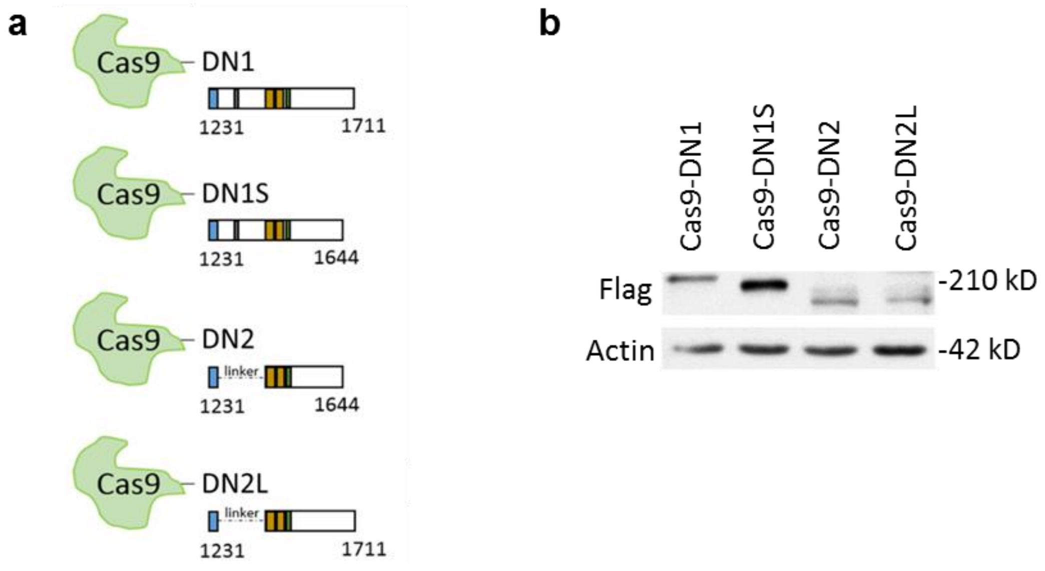

Supplementary Figure 4. Fusion of Cas9 to selected 53BP1 dominant negative sequences.
a. Schematic diagram showing the different truncated 53BP1 proteins (DN1, DN1S, DN2 and DN2L) fused with Cas9. **b**. The relative expression levels of the different Cas9 fusion proteins were analyzed by western blot using anti-FLAG antibody and anti-actin antibody (loading control) in HeLa cells transfected with plasmids encoding a FLAG-tagged Cas9 fusion to DN 53BP1 (DN1, DN1S, DN2 or DN2L) and GFP. Each arm was sorted for GFP+ cells (DN transduced cells) prior to western blot analysis. Cas9 denotes SpCas9. Source data is available in Source Data file.

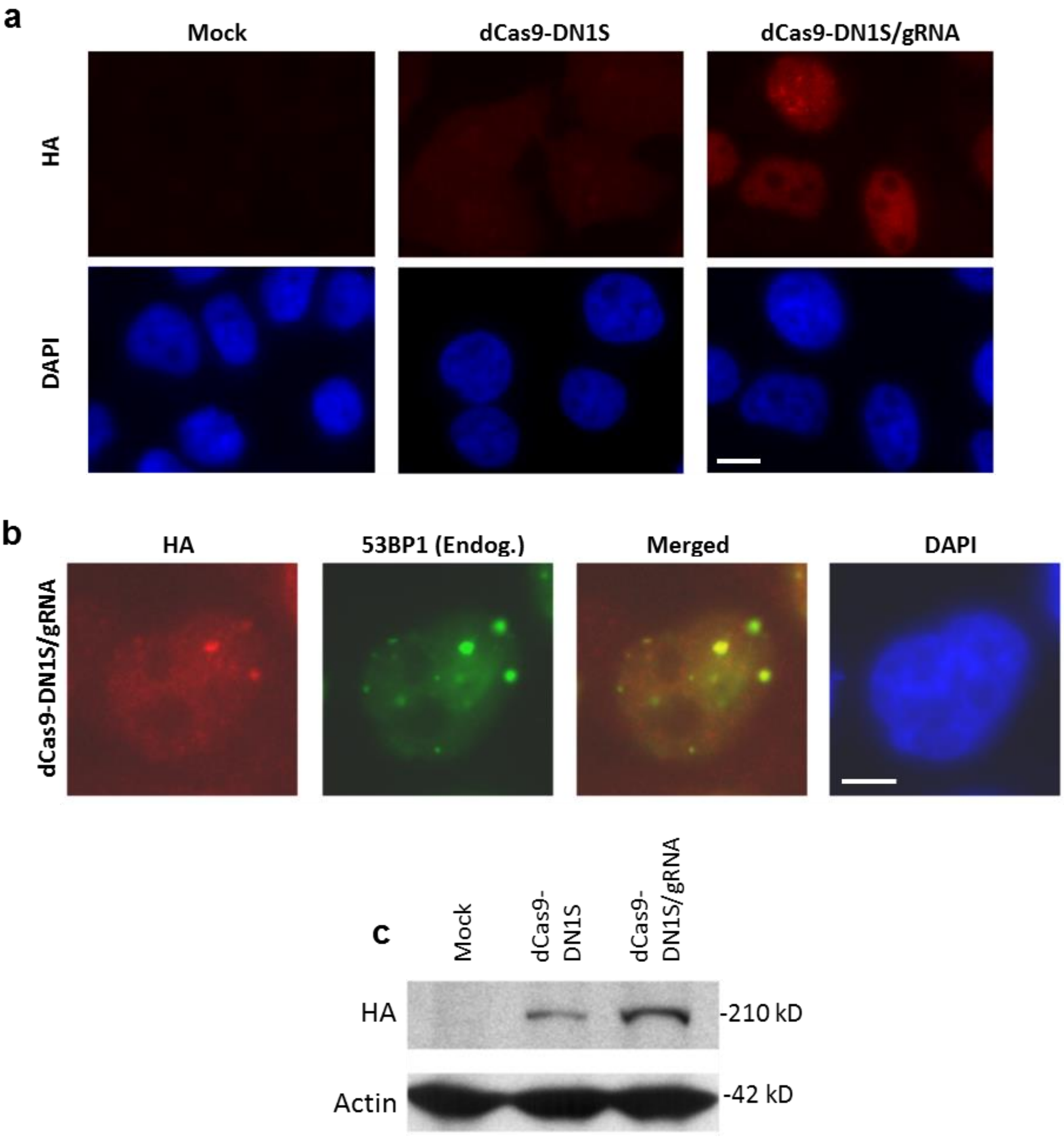

Supplementary Figure 5. A specific gRNA is necessary to stabilize and target dCas9-DN1S to nuclei; in the presence of gRNA, dCas9-DN1S fusion protein is only occasionally co-localized with endogenous 53BP1 in foci. a. Representative immunofluorescence (IF) images showing the presence or absence of HA-tagged dCas9-DN1S or dCas9-DN1S/gRNA in nuclei. **b.** Co-localization of dCas9-DN1S/gRNA (HA) with endogenous 53BP1 foci is shown. **c.** An immunoblot shows the levels of HA-tagged dCas9-DN1S with and without CD45 gRNA, detected with anti-HA antibodies. Anti-actin is shown as a loading control. For panels a and b, scale bars represent 10 μm. In panels a-c: dCas9 denotes catalytically dead SpCas9. Source data is available in Source Data file.

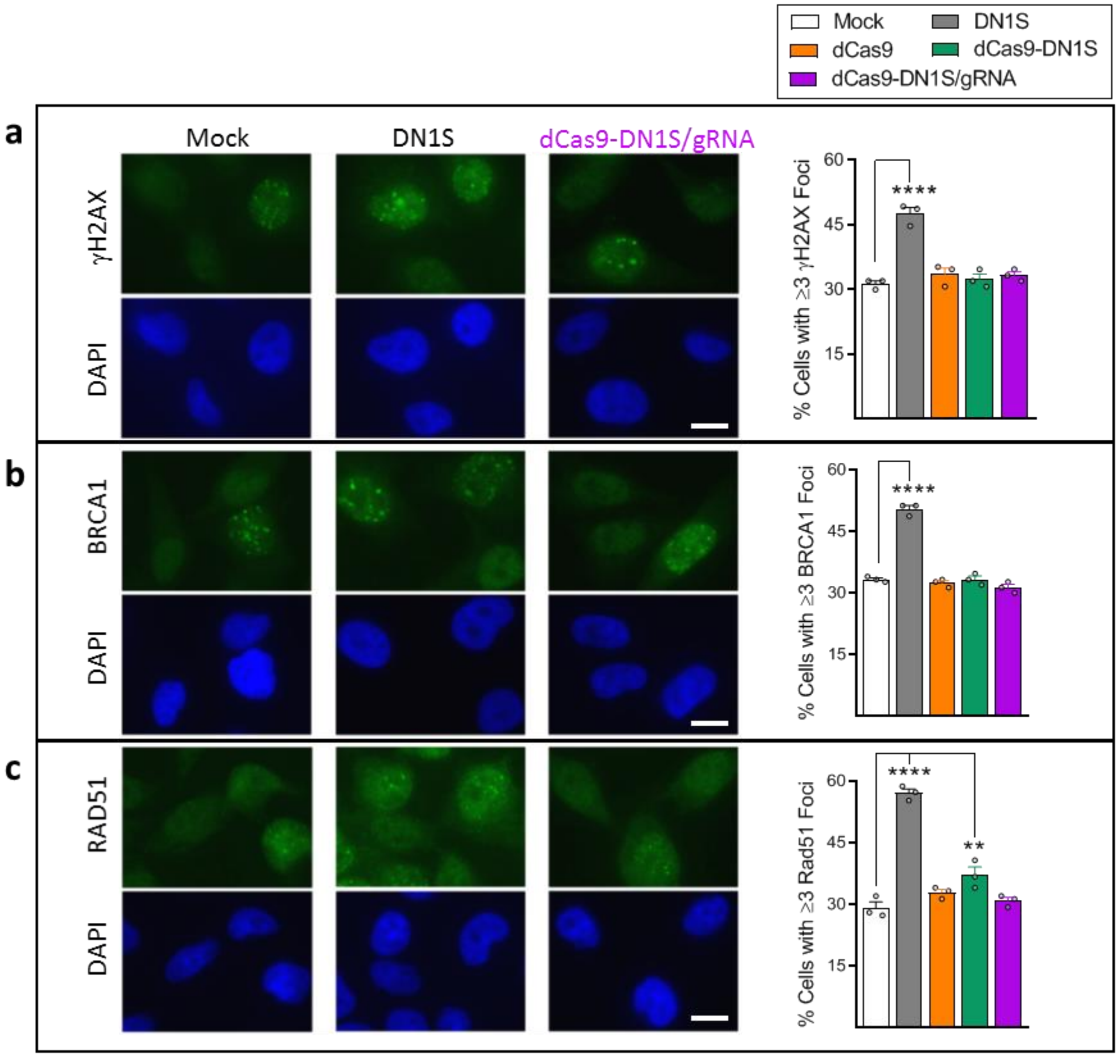

Supplementary Figure 6. Untethered DN1S, but not dCas9-DN1S (with or without gRNA) increases γH2AX, BRCA1 and RAD51 nuclear foci. Representative immunofluorescence (IF) images of HeLa cells labeled for **(a)** γH2AX**, (b)** BRCA1, and **(c)** RAD51 with lentiviral expression of DN1S, dCas9 and dCas9-DN1S, with and without gRNA. Scale bars represent 20 μm. Quantification of all groups tested with ≥3 γH2AX, BRCA1 or RAD51 foci is presented as the mean ± SEM of three counts of 150 cells or more each in three independent fields, adjacent to the respective IF images. Black circles indicate individual counts. Statistics for all panels: ANOVA. Only significant differences are marked: ** indicates $p<0.01$ and **** indicates $p<0.0001$. For all panels: dCas9 denotes catalytically dead SpCas9. Source data is available in Source Data file.

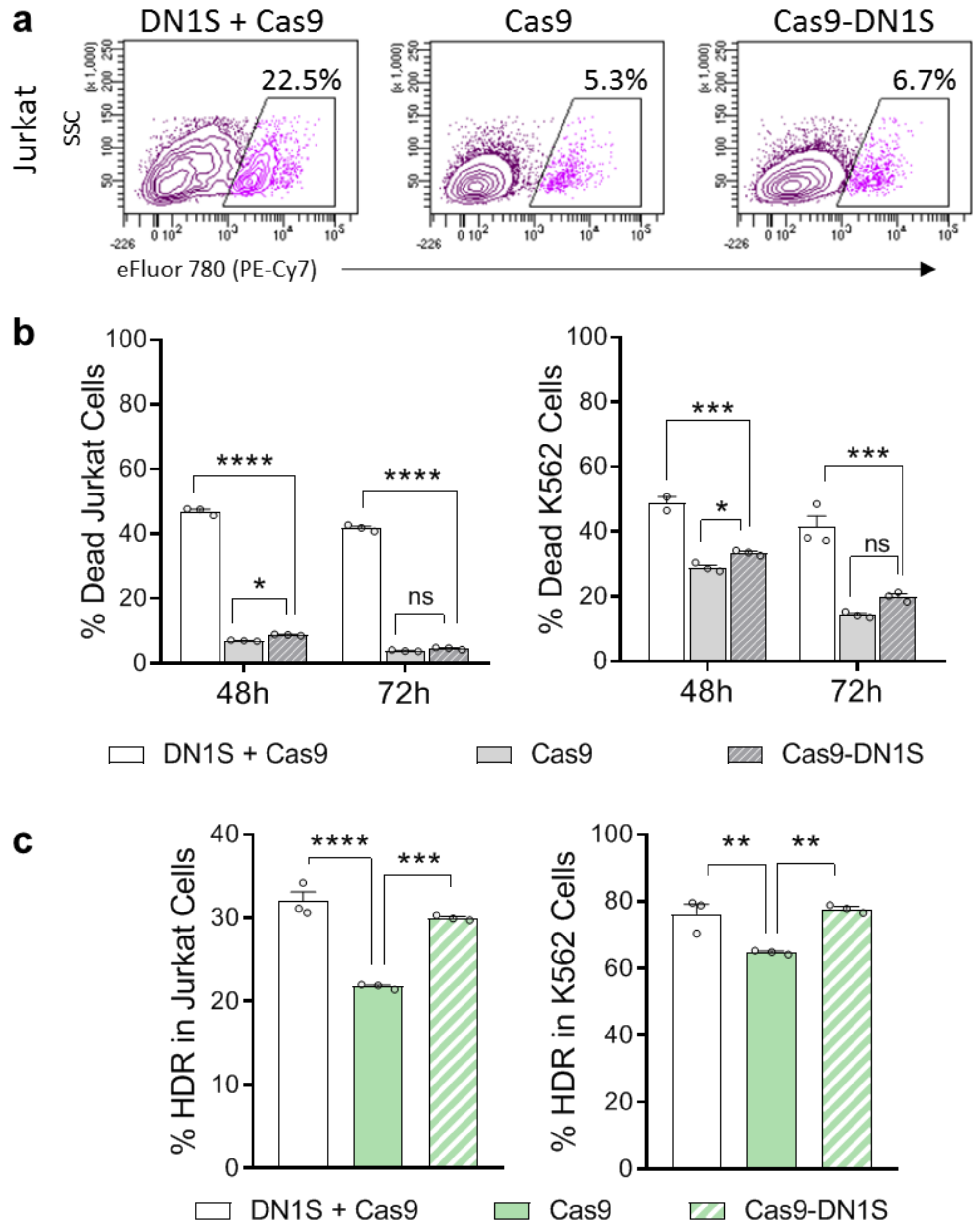

Supplementary Figure 7. Untethered DN1S plus Cas9 RNP has increased acute cellular toxicity over Cas9 RNP and Cas9-DN1S, but increases HDR frequency similar to Cas9-DN1S RNP.
a. Representative FACS plots demonstrating non-viable Jurkat cells with lentiviral-expressed DN1S plus Cas9 RNP, Cas9 RNP only, or Cas9-DN1S RNP using eFluor 780 fixable viability dye at 24 hours after electroporation. Quantitative data is shown in Figure 2f. **b.** Viability at 48 hours and 72 hours post-electroporation and rAAV6 GFP donor delivery in Jurkat and K562 cells as measured by eFluor 780 fixable viability dye. **c.** Quantification of HDR-mediated editing at the CD45 locus in Jurkat cells, and the AAVS1 locus in K562 cells. HDR was quantified as the percentage of GFP+ cells 14 days after RNP electroporation and rAAV6 GFP donor delivery. Panels b and c: Data are presented as the mean ± SEM of three independent electroporations, except in the DN1S + Cas9 group of K562 cells at 48 hours in panel b, which shows only two independent electroporations. Black circles indicate individual data points. Statistics for panels b and c: ANOVA; ns indicates not significant, * indicates p<0.5, ** indicates p<0.01 , *** indicates p<0.001, and **** indicates p<0.0001. Source data is available in Source Data file.

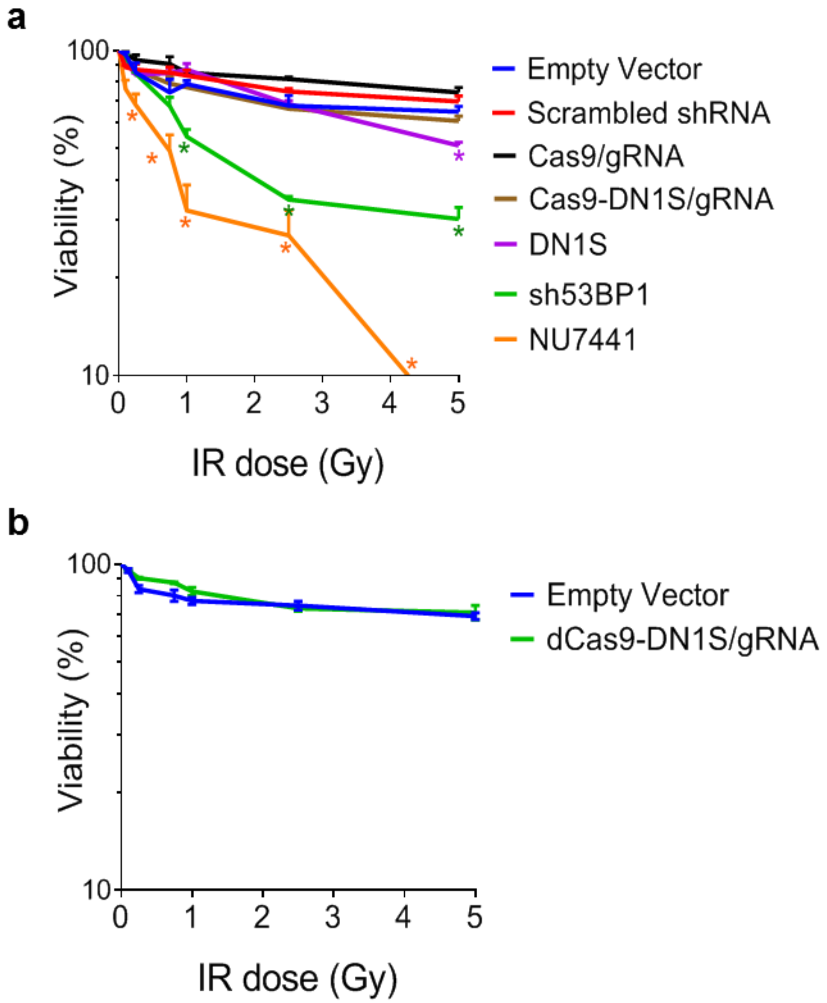

Supplementary Figure 8. Unlike global inhibition of NHEJ (NU7441, sh53BP1 or untethered DN1S), Cas9-DN1S does not increase cellular sensitivity to IR. **a**. HeLa cells stably transduced with lentivirus constructs or exposed to NU7441, were treated with IR at the indicated doses. Viability was determined by crystal violet staining and colony counts. Global NHEJ inhibition (sh53BP1 or NU7441) resulted in decreased cell viability in response to IR, as compared with Cas9 or Cas9-DN1S with gRNA and controls. For each condition, values are normalized to those of untreated controls. The data are presented as the mean ± SEM of three independent transductions. * indicates $p<0.05$ as determined by unpaired two-tailed t tests. Cas9 denotes SpCas9. **b.** HeLa cells transduced with catalytically dead Cas9 (dCas9) or control were treated with IR at the indicated doses. Viability was determined by crystal violet staining and colony counts. For each condition, values are normalized to those of untreated controls. The data are presented as the mean ± SEM of three independent transductions. Here, dCas9 denotes dSpCas9. Source data is available in Source Data file.

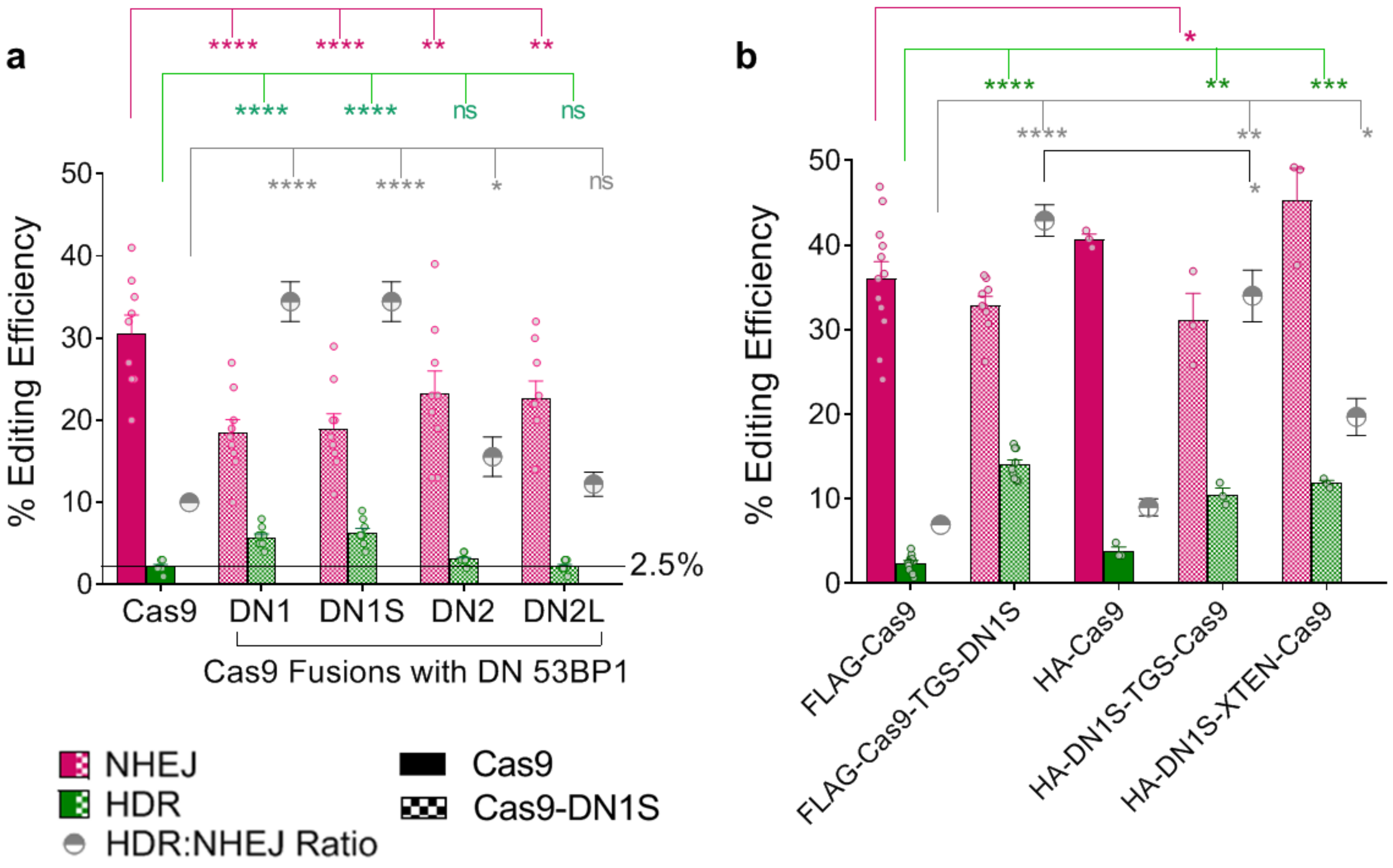

Supplementary Figure 9. Cas9 fused to DN1 and DN1S increase HDR and decrease NHEJ with optimal effect in the Flag-SpCas9-TGS-DN1S configuration. **a.** Relative frequencies of HDR (green bars) and NHEJ (magenta bars), and the HDR/NHEJ ratio (grey half circles) induced by different SpCas9 fusion proteins (shaded bars) were compared to those induced by SpCas9 alone (solid bars) using the previously published TLR system in 293T cells. The TLR system comprises the exogenous Rosa26 locus integrated into 293T cells at the endogenous AAVS1 locus. All constructs were delivered via transfection of the nuclease plasmids along with the Venus donor plasmid. HDR was detected by quantifying percent Venus+ cells, and NHEJ was detected by quantifying percent RFP+ cells. The data are presented as the mean ± SEM of nine independent transfections. Circles indicate individual data points. The black line marks the mean % HDR in Cas9 controls. **b**. Relative frequencies of HDR (green bars) and NHEJ (magenta bars), and the HDR/NHEJ ratio (grey half circles), induced by SpCas9-DN1S fusion protein with different epitope tags and linkers were compared to those induced by SpCas9 using the TLR system in 293T cells. All nuclease constructs and the Venus donor were delivered as plasmid transfections. The data are presented as the mean ± SEM of 3-12 transfections. Circles indicate individual data points. Statistics for a and b: ANOVA comparing NHEJ or HDR, or HDR/NHEJ ratios: ns indicates not significant, * indicates $p<0.05$, ** indicates $p<0.01$, *** indicates $p<0.001$, and **** indicates $p<0.0001$. Cas9 denotes SpCas9 in both panels. Source data is available in Source Data file.

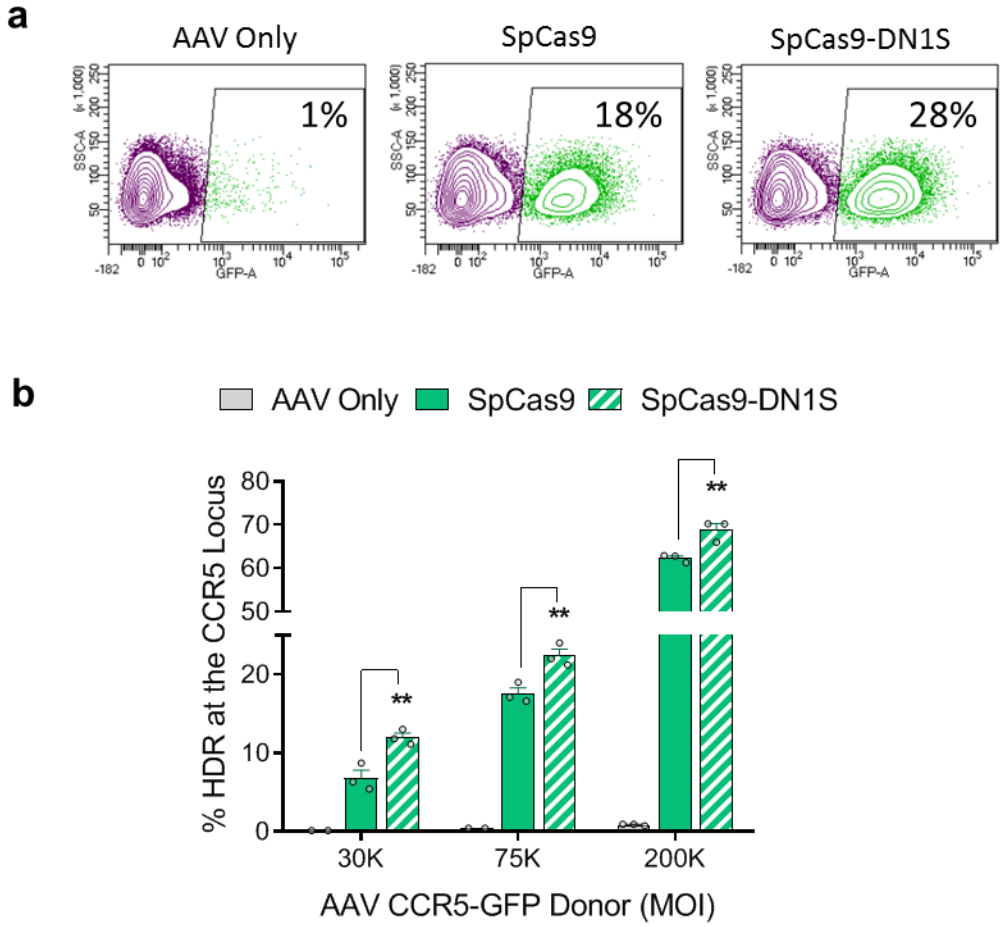

Supplementary Figure 10. SpCas9-DN1S increases targeted knock-in of GFP at the CCR5 locus in Jurkat T cells. **a.** Representative flow cytometry plots of GFP knock-in at the CCR5 locus in Jurkat cells by electroporation of SpCas9 or SpCas9-DN1S RNP with CCR5-GFP rAAV6 donor provided at an MOI of 120K. Quantification is shown in Figure 3a. **b.** Quantification of HDR at the CCR5 locus in Jurkat cells with SpCas9 or SpCas9-DN1S RNP and CCR5-GFP rAAV6 donor of variable MOI. HDR was quantified as the percentage of GFP+ events by flow cytometry. Black circles indicate individual data points. The data are presented as the mean ± SEM of three independent electroporations, except in the AAV only groups of 30K and 75K, where only two replicates were done. Statistics: unpaired t tests, one tailed. ** indicates $p<0.01$. Source data is available in Source Data file.

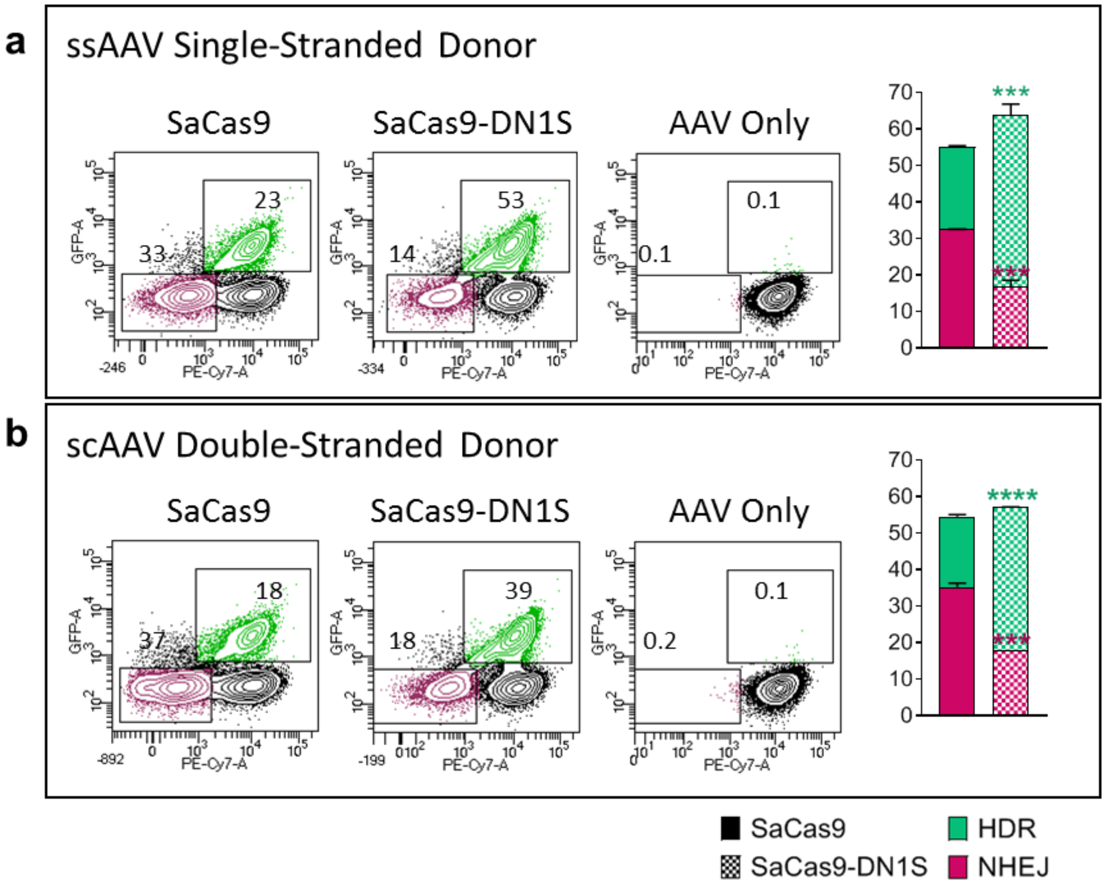

Supplementary Figure 11. SaCas9-DN1S increases HDR and decreases NHEJ with both ssAAV and scAAV donor templates. Representative flow cytometry plots and stacked bar plot quantification of editing events at the CD45 locus in Jurkat cells with SaCas9 or SaCas9-DN1S RNP and **(a)** single-stranded ssAAV6 donor or **(b)** double-stranded scAAV6 donor delivered at an MOI of 200K. HDR was quantified as percentage of CD45+GFP+ events by flow cytometry. NHEJ was quantified as percentage of CD45- events by flow cytometry. CD45 is indicated by PE-Cy7 on the X axis. With the high HDR efficiency, the pattern of GFP+ Jurkat cell population shows a higher density of GFP+ cells and likely represents more bi-allelic HDR in the SaCas9-DN1S edited cells. The data are presented as the mean ± SEM of three independent electroporations. Statistics: unpaired t tests, one tailed. * indicates $p<0.05$ *** indicates $p<0.001$ and **** indicates $p<0.0001$. Source data is available in Source Data file.

a
b

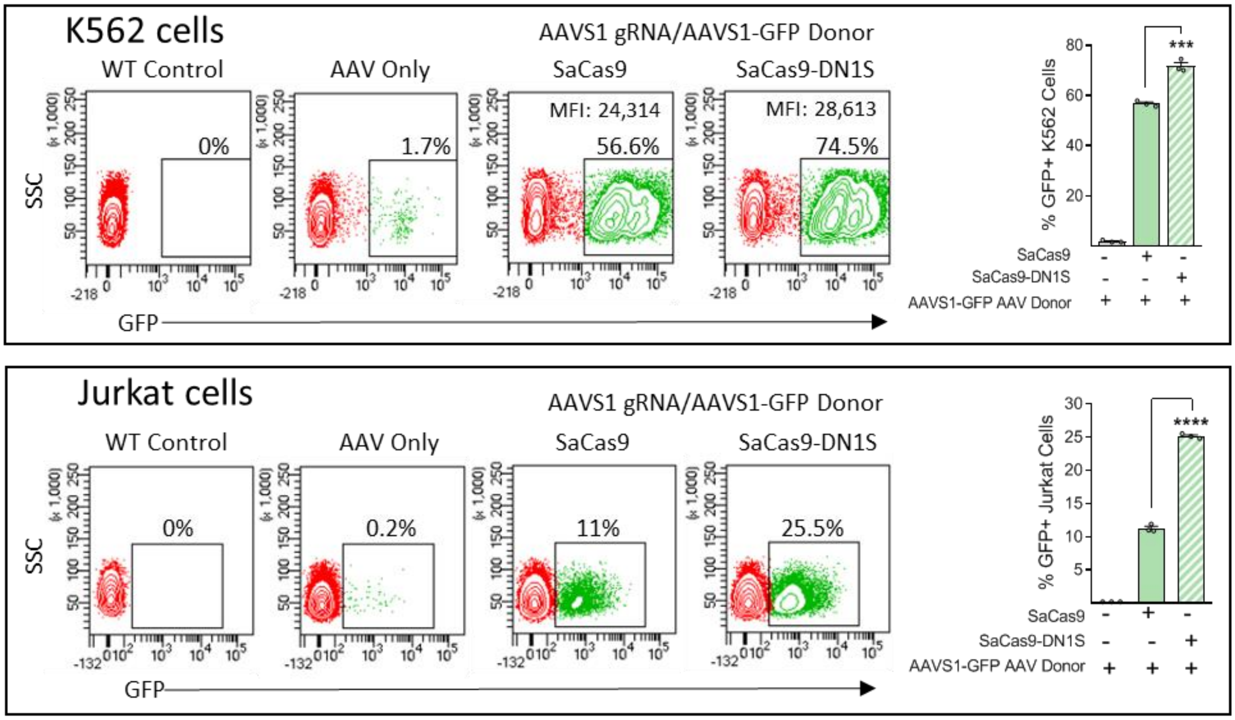

c

Cell Line	RNP	GFP+ Clones	5′ HDR+ Clones	% HDR in GFP+ Clones	% RI in GFP+ Clones
K562	SaCas9	45	43	96	4
	SaCas9-DN1S	47	46	98	2
Jurkat	SaCas9	50	40	80	20
	SaCas9-DN1S	47	45	96	4

Supplementary Figure 12. Cas9-DN1S increases HDR efficiency without increasing off-target integration of the AAV donor template. a. Representative flow cytometry plots and quantification of HDR at the AAVS1 locus in K562 cells with SaCas9 or SaCas9-DN1S and AAVS1-GFP donor delivered via rAAV6. HDR was quantified as the percentage of GFP+ cells 14 days after electroporation and AAV donor delivery. With high HDR efficiency, the density of the $GFP^{bright+}$ population is greater in cells edited by SaCas9-DN1S, reflected by the mean fluorescence intensity (MFI), as noted. **b.** Representative flow cytometry plots and quantification of HDR at the AAVS1 locus in Jurkat cells with SaCas9 or SaCas9-DN1S and AAVS1-GFP rAAV6 donor. HDR was quantified as the percentage of GFP+ cells 14 days after electroporation and AAV donor delivery. For panels a and b: The data are presented as the mean ± SEM of three independent electroporations. Black circles indicate individual data points. Statistics: unpaired t tests, one tailed. *** indicates $p<0.001$ and **** indicates $p<0.0001$. **c.** Molecular confirmation of single-cell colonies for correct on-target integration at the AAVS1 locus. All colonies were tested for ApoB and GFP to ensure DNA quality and presence of the donor GFP, and further tested for the presence of 5’ HDR at AAVS1 by PCR. Colonies demonstrating amplification of both GFP and 5’ HDR were marked as on-target. Colonies demonstrating amplification of GFP without amplification of 5’ HDR were considered to have off-target random integration (RI). Source data is available in Source Data file.

a

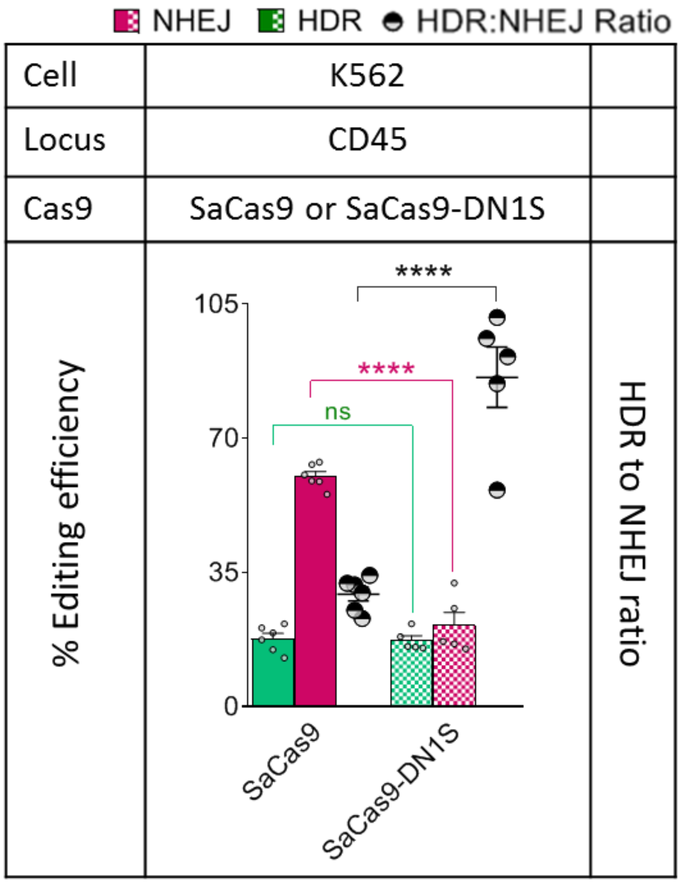

Supplementary Figure 13. SaCas9-DN1S significantly decreases NHEJ with modest effects on HDR in K562 cells at the CD45 locus. **a**. Relative frequencies of HDR, NHEJ (magenta bars and asterisks) and the HDR/NHEJ ratio (black half circles and asterisks) induced by SaCas9 or SaCas9-DN1S RNP and CD45-GFP rAAV6 donor template in K562 cells. Small black circles indicate individual data points. The data are presented as the mean ± SEM of 5-6 independent electroporations. Statistics: unpaired t tests, one tailed. **** indicates p<0.0001. Source data is available in Source Data file.

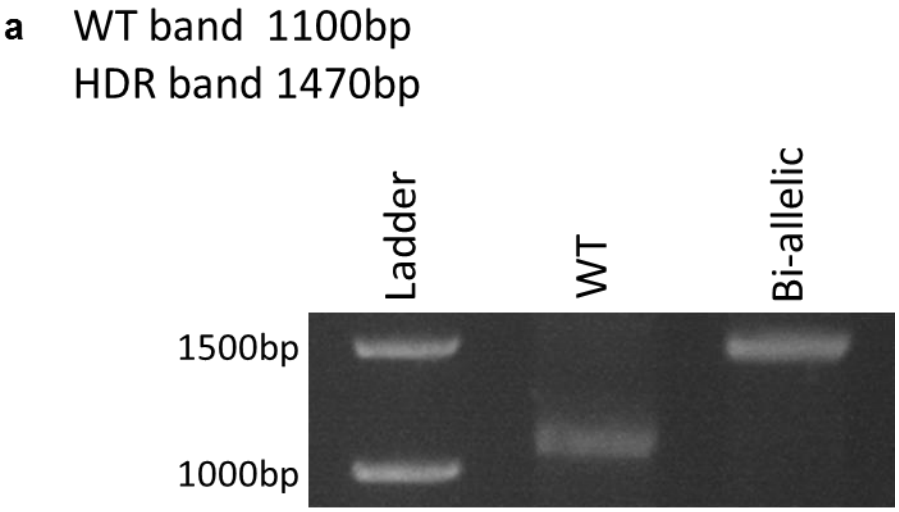

Supplementary Figure 14. Cas9-DN1S increases bi-allelic editing events.
a. Gel image of PCR bands of unedited (WT) cells and edited cells sorted from high CD18 expression (bi-allelic editing). Source data is available in Source Data file.

a

gRNA	Target ID	Target Sequence (SpCas9)	PAM
AAVS1	On target	G G G G C C A C T A G G G A C A G G A T	TGG
AAVS1	OT14	G G G A C C A T C A G G G A C A G G A T	TGG
AAVS1	OT7	G G G G C C C C T G G G G A C A G A A T	TGG
AAVS1	OT11	G G G G C C A A T T A G G A C A G G A T	TGG
AAVS1	OT13	G G G G C T T C T A A G G A C A G G A T	GGG

b

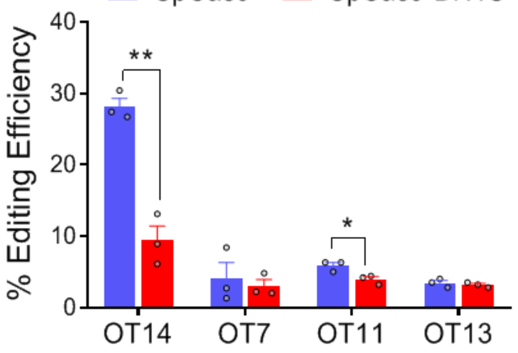

c

gRNA	Target ID	Target Sequence (SaCas9)	PAM
AAVS1	On target	C A C A G T G G G G C C A C T A G G G A	CAGGA
AAVS1	OT1	G A C A T T G G G G C C T C T A C G T A	CTGAA
AAVS1	OT2	C A C T C T G G A G C C A C T A G G G A	AGGGG
AAVS1	OT3	C A C A G T G A G T C A A C T A G G G A	GTGGG
AAVS1	OT4	C A C C G T G G G G C C C C T A G G G G	AAGGG

d

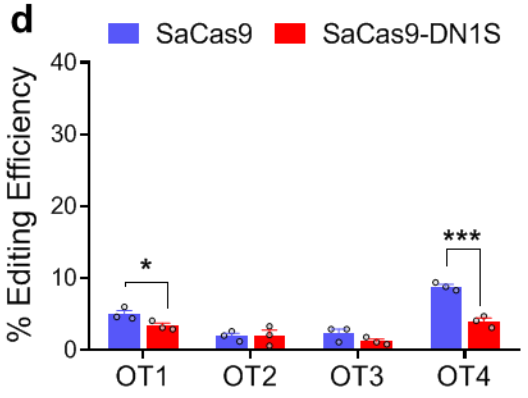

e

gRNA	Target ID	Target Sequence	PAM
CD45	On target	G A A A G G C A A A G C C A A A T G C C	AAGAG
CD45	OT2	G A A A G A C A A A G C C A A A T G C T	TTGAA
CD45	OT3	G A A A C C C A A A G A C A A A T G C C	CTGGA
CD45	OT4	T A G A A G C A A A G C C A A T T G C C	AGGAA

f

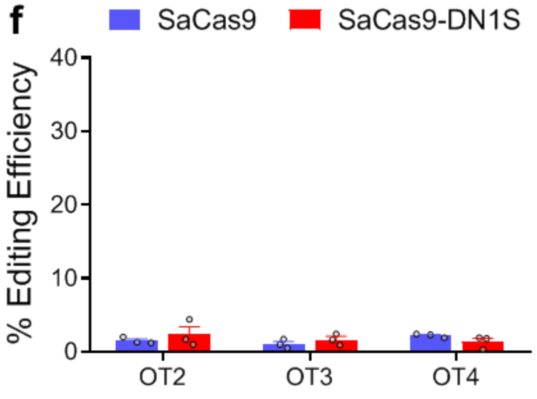

Supplementary Figure 15. SpCas9-DN1S or SaCas9-DN1S do not increase the NHEJ efficiency at the tested off-target sites. a. SpCas9 AAVS1 off-target sequences compared to on-target SpCas9 AAVS1 gRNA. Base mismatches in the off-target sequences are highlighted in red. **b.** NHEJ editing efficiency of SpCas9 or SpCas9-DN1S at selected off-target (OT) sites of AAVS1 SpCas9 gRNA in LAD patient-derived B lymphocytes. **c.** Predicted SaCas9 AAVS1 off-target sequences compared to on-target SaCas9 AAVS1 gRNA. Base mismatches in the off-target sequences are highlighted in red. **d.** NHEJ editing efficiency of SaCas9 or SaCas9-DN1S at selected off-target sites of AAVS1 SaCas9 gRNA in LAD patient-derived B lymphocytes. **e.** Predicted SaCas9 CD45 off-target sequences compared to on-target SaCas9 CD45 gRNA. Base mismatches in the off-target sequences are highlighted in red. **f.** NHEJ editing efficiency of SaCas9 or SaCas9-DN1S at selected off-target sites of CD45 SaCas9 gRNA in LCL cells. For panels b, d, and f: The data are presented as the mean ± SEM of three independent electroporations. Black circles indicate individual data points. Statistics: unpaired t tests, one tailed comparing Cas9 to Cas9-DN1S: * indicates $p<0.05$, ** indicates $p<0.01$, and *** indicates $p<0.001$, only significant groups are indicated. Source data is available in Source Data file.

Supplementary Tables

Supplementary Table 1. gRNA sequences.

Target	Recognition Sequence	PAM	Cas9 System
CD45	GAG TTT AAG CCA CAA ATA CA	TGG	SpCas9 or dSpCas9 by Plasmid or Lentivirus
CENP-B	AGA AAT CCC GTT TCC AAC GA	AGG	dSpCas9 by Lentivirus
Rosa26	ACT CCA GTC TTT CTA GAA GA	TGG	SpCas9 in TLR-293T
AAVS1	GGG GCC ACT AGG GAC AGG AT	TGG	SpCas9 by Plasmid
LMO2	TCG TGA AGT CAG GGC TTC TA	AGG	SpCas9 by Plasmid
CCR5	GCA GCA TAG TGA GCC CAG AA	GGG	SpCas9 by RNP
AAVS1	CAC AGT GGG GCC ACT AGG GA	CAGGAT	SaCas9 by RNP
CD45	GAA AGG CAA AGC CAA ATG CC	AAGAGT	SaCas9 by RNP

Supplementary Table 2. Primers used for generating dominant negative 53BP1 constructs.

1230-START-FW	gaa cag cac ggc gca gat ata cc
1230-START-RV	cat ggc cat gtg gca tgg cgt agt c
1480-START-FW	ctc gag taa tta gtt tga ttt taa cct agg ttc
1480-START-RV	cta ttt cct gga gag gac atg gcg tag
1644-END-FW	gag atc taa cgt cag ctc ccc agc c
1644-END-RV	ctg cag tcg acg ggc ccg gg
1711-END-FW	cgg aga caa cac cgg gga gta att ag
1711-END-RV	cta tga cca tga tta cgc caa gct tgc
1230-FW	cca cat ggc cat gtc tta cat cgt cac
1480-FW	cct ctc cag gaa ata gct ttg tag gg
1644-RV	gct cga gga ggc agt agg ggt g
1711-RV	cgg tgt tgt ctc cgg act cac agg

Supplementary Table 3. Primers used for generation of Cas9-DN fusion constructs.

Cas9-SanDI-FW	gct gat cgc cag aaa gaa gga ctg gg
Cas9-RV	gac cct gtg tcg cct ccc agc tg
Cas9-53BP1-Fusion-FW	ggc gac aca ggg tcc aca gga tc
53BP1-1480-FW	cca gga aat agc ttt gta ggg ctc cg
53BP1-1644-RV	cct ttt gct cga gga ggc agt agg
53BP1-1711-RV	ggc ctt ttc tcc ccg gtg ttg tct cc
Cas9-NLS-FseI-RV	ctg ccg aat tcc ttt ttc ttt ttt gcc tgg
Cas9-NLS-FseI-Oligo	aaaaggccggcggccacgaaaaaggccggccaggc aaaaaagaaaaaggaattcggcag
53BP1-1230-Linker1	ggcgacacagggtccacaggatccacaggcagcac agggagcatgggaccacatggccatgtcttacatcgt cacatgagaacaatccgggaagtacgcacacttgtc actcgtgtcattacagatg
53BP1-1230-Linker2	gtgtcattacagatgtgtattatgtggatggaacagaag tagaaagaaaagtaactgaggagactgaagagcca attacagggtccacaggatccacaggcagcacagg gagcatgggatcctctccaggaaatagc
P153-Cas9	gat cac aaa gca cgt ggc aca g
53BP1-1644-SacII-RV	tcg ttt cgc cgc ggg gct cga gga gg
ZSpCas9-D10A-FW	agc atc ggc ctg gcc atc ggc acc aac
ZSpCas9-D10A-RV	gtt ggt gcc gat ggc cag gcc gat gct
ZSp-N863A-FW	cca gaa gcg aca agg ccc ggg gca aga gcg
ZSp-N863A-RV	cgc tct tgc ccc ggg cct tgt cgc ttc tgg

Supplementary Table 4. Primers used for TIDE assay.

Region	Primers	Size
AAVS1	F: gag aaa ggg agt aga ggc gg	986 bp
	R: tgg aca acc cca aag tac cc	
	Seq: ggg tca cct ctc act cct ttc	
CD45	F: gtc atc ttg cca aca ccc att	728 bp
	R: agc agg ctt ctc act tcc agt t	
	Seq: tca tca cct agc agt tca tgc ag	
SaCas9 AAVS1 CSOT	F: aaa gca atg ggt tta tgg cgg g	975 bp
	R: ggc aag ggg gag ctt ttt gtc	
SaCas9 AAVS1 OT2	F: gtg att ctc agg cac agg gtc t	791 bp
	R: cag tct agt tca cag gag ctg gt	
SaCas9 AAVS1 OT3	F: cat ttg gcc taa gac ccc tcc a	637 bp
	R: tct cga cac gca gaa gac aga g	
SaCas9 AAVS1 OT4	F: aac atc aag ttt gcc gac cag c	915 bp
	R: ctc ctg gac cac ata agc cac a	
SpCas9 AAVS1 OT7	F: ctg gtg tgc tga gga cct tt	1071 bp
	R: ccc atc cta gac aca ggg ga	
	Seq: ctc tct tca tac aac cac caa g	
SpCas9 AAVS1 OT11	F: tgc tta gtg cat tgg ccg ta	1104 bp
	R: acc tcc acc ttc cca tcc t	
	Seq: gac aca gca ggg agt ttt gg	
SpCas9 AAVS1 OT13	F: att ggt tgt ctt gcc cca ct	741 bp
	R: cct cca tgc cca cca ttt ct	
	Seq: cca ctg gga tga gtc atg tc	
SpCas9 AAVS1 OT14	F: cca gtg gga act tgg cag aa	788 bp
	R: cat gcc tca ctg ctc tca ca	
	Seq: tcc cag tct gcc ctc tct tt	
SaCas9 CD45 OT2	F: ggg cag agg gtg att tca gga t	683 bp
	R: tga tgg cag gaa gaa gga gag c	
SaCas9 CD45 OT3	F: gga tca aga ctt ccc cac tgc t	843 bp
	R: agg ctt gcc ttg cta ccc ata t	
SaCas9 CD45 OT4	F: aac act gga cat cgg tct cct g	812 bp
	R: gtc aca cca gcg aat gtc agt g	

Supplementary Table 5. Primers and program conditions for random integration qPCR.

**ApoB/GFP Primers**	**ApoB/GFP Program**
hApoB FW: ctt ggt tta tga atc tgg ctc	95°C, 3 min<br>{95°C, 5 sec; 60°C, 30 sec} x40<br>4°C, hold
hApoB RV: gcc ttt agc agt tag aac ac	
hApoB HEX Probe: atc aca agt cga ttc cca gca tgt	
GFP FW: act aca aca gcc aca acg tct ata tca	
GFP RV: ggc gga tct tga agt tca cc	
GFP FAM Probe: ccg aca agc aga aga acg gca tca	
**5' HDR Primers**	**5' HDR Program**
AAVS1 FW: ccg tct tcc tcc act ccc tc	95°C, 5 min<br>{95°C, 5 sec; 62°C, 60 sec} x40<br>Melt curve:<br>65°C to 95°C, 5 sec, +0.5°C per cycle<br>4°C, hold
MND RV: gac cac tga tat cct gtc ttt aac	

Supplementary Table 6. Primers and PCR program for WT/NHEJ vs HDR

**WT/NHEJ vs. HDR Primers**	**WT/NHEJ vs. HDR Program**
AAVS1-WT-FW: caa agt acc ccg tct ccc tg	95°C, 5 min<br>{98°C, 20 sec; 66°C, 15 sec; 72°C, 1 min} x35<br>4°C, hold
AAVS1-WT-RV: gcc aag gac tca aac cca ga	
AAVS1-HR-FW3: ctg tgg caa gta cat ctc ctg	
