# Supplementary material for: CRISPR-Cas9 fusion to dominant-negative 53BP1 enhances HDR and inhibits NHEJ specifically at Cas9 target sites
Source: Nat Commun. 2019 Jun 28;10:2866. doi: 10.1038/s41467-019-10735-7 (PMC6598984; doi:10.1038/s41467-019-10735-7)
Supplement: Supplementary file 2 — Reporting Summary [file 41467_2019_10735_MOESM2_ESM.pdf]

nature research

Corresponding author(s): Punam Malik

Last updated by author(s): May 22, 2019

Reporting Summary

Nature Research wishes to improve the reproducibility of the work that we publish. This form provides structure for consistency and transparency in reporting. For further information on Nature Research policies, see Authors & Referees and the Editorial Policy Checklist.

Statistics

For all statistical analyses, confirm that the following items are present in the figure legend, table legend, main text, or Methods section.

n/a	Confirmed	
☐	☒	The exact sample size (*n*) for each experimental group/condition, given as a discrete number and unit of measurement
☐	☒	A statement on whether measurements were taken from distinct samples or whether the same sample was measured repeatedly
☐	☒	The statistical test(s) used AND whether they are one- or two-sided<br>*Only common tests should be described solely by name; describe more complex techniques in the Methods section.*
☒	☐	A description of all covariates tested
☐	☒	A description of any assumptions or corrections, such as tests of normality and adjustment for multiple comparisons
☐	☒	A full description of the statistical parameters including central tendency (e.g. means) or other basic estimates (e.g. regression coefficient) AND variation (e.g. standard deviation) or associated estimates of uncertainty (e.g. confidence intervals)
☐	☒	For null hypothesis testing, the test statistic (e.g. *F*, *t*, *r*) with confidence intervals, effect sizes, degrees of freedom and *P* value noted<br>*Give P values as exact values whenever suitable.*
☒	☐	For Bayesian analysis, information on the choice of priors and Markov chain Monte Carlo settings
☒	☐	For hierarchical and complex designs, identification of the appropriate level for tests and full reporting of outcomes
☒	☐	Estimates of effect sizes (e.g. Cohen's *d*, Pearson's *r*), indicating how they were calculated

Our web collection on statistics for biologists contains articles on many of the points above.

Software and code

Policy information about availability of computer code

Data collection	Not applicable
Data analysis	Graph Pad Prism version 8, Microsoft Excel version 2013, Deskgen (TIDE at https://tide.deskgen.com/)

For manuscripts utilizing custom algorithms or software that are central to the research but not yet described in published literature, software must be made available to editors/reviewers. We strongly encourage code deposition in a community repository (e.g. GitHub). See the Nature Research guidelines for submitting code & software for further information.

Data

Policy information about availability of data

All manuscripts must include a data availability statement. This statement should provide the following information, where applicable:

- Accession codes, unique identifiers, or web links for publicly available datasets
- A list of figures that have associated raw data
- A description of any restrictions on data availability

The authors declare that the data supporting the findings of this study are
available within the paper and its supplementary information files. Data availability statement and all raw data associated with the figures is included.

Field-specific reporting

Please select the one below that is the best fit for your research. If you are not sure, read the appropriate sections before making your selection.

☒ Life sciences ☐ Behavioural & social sciences ☐ Ecological, evolutionary & environmental sciences

Life sciences study design

All studies must disclose on these points even when the disclosure is negative.

Sample size	We routinely do at least 3 or more biological replicates per experiment. No power analysis and sample size detemination is necessary in these type of studies
Data exclusions	We did not exclude any data points from the experiments presented. These data do not include initial experiments done to optimize editing parameters (electroporation parameters, plasmid or RNP complex formation, AAV production to optimal titers, etc). Once optimal editing platform was developed, experiments were replicated at least three or more times.
Replication	We have replicated experiments three times at minimum. Where findings were unexpected (Fig. S12), more replicates were done to confirm results.
Randomization	Not applicable
Blinding	There was no data blinding

Reporting for specific materials, systems and methods

We require information from authors about some types of materials, experimental systems and methods used in many studies. Here, indicate whether each material, system or method listed is relevant to your study. If you are not sure if a list item applies to your research, read the appropriate section before selecting a response.

Materials & experimental systems

n/a	Involved in the study
☐	☒ Antibodies
☐	☒ Eukaryotic cell lines
☒	☐ Palaeontology
☒	☐ Animals and other organisms
☒	☐ Human research participants
☒	☐ Clinical data

Methods

n/a	Involved in the study
☒	☐ ChIP-seq
☐	☒ Flow cytometry
☒	☐ MRI-based neuroimaging

Antibodies

Antibodies used

Antibodies for IF:
53BP1: Rabbit Polyclonal Anti-53BP1 Antibody, Novus Biologicals, catalog #NB100-304, Lot E-1
https://www.novusbio.com/products/53bp1-antibody_nb100-304

Actin: Mouse Anti-Human Actin, Santa Cruz, catalog #sc-8432, Clone C-2, Lot #F0413
https://www.scbt.com/scbt/product/actin-antibody-c-2

HA: Mouse Monoclonal HA Antibody, Covance, catalog #16B2, Clone HA.11, Lot #14860501
https://www.biolegend.com/en-us/products/purified-anti-ha-11-epitope-tag-antibody-11374

FLAG: Mouse Monoclonal FLAG Antibody, Sigma, catalog #F1804, Clone M2, no lot number given
https://www.sigmaaldrich.com/catalog/product/sigma/f1804?lang=en&region=US

yH2AX: Anti-phospho-histone H2A.X (Ser139), EMD Millipore, catalog #05-636, Clone JBW301, Lot #3103591
http://www.emdmillipore.com/US/en/product/Anti-phospho-Histone-H2A.X-Ser139-Antibody-clone-JBW301,MM_NF-05-636

CENP-B: Rabbit Anti-Human CENP-B Antibody, Active Motif, catalog #61288, no clone number, no lot number
https://www.activemotif.com/catalog/details/61287/cenp-b-antibody-pab-2

Rad51: Rabbit Polyclonal Rad51 Antibody, Santa Cruz, catalog #sc-8349, Clone H-92, Lot # J0215
https://www.scbt.com/scbt/product/rad51-antibody-h-92

BRCA1: Mouse Monoclonal Anti-Human BRCA1, Santa Cruz, catalog #sc-6954, Clone D-9, Lot #D1713
https://www.scbt.com/scbt/product/brca1-antibody-d-9

BRCA1: Rabbit Anti-BRCA1 Antibody, Cell Signaling, catalog #9010, Lot 1
https://www.cellsignal.com/products/primary-antibodies/brca1-antibody/9010

RIF1: Rabbit Polyclonal Anti-Human RIF1 Antibody, Novus Biologicals, catalog #NBP2-47303, Lot R34461
https://www.novusbio.com/products/rif1-antibody_nbp2-47303

	Antibodies for flow cytometry:<br>PE-Cy7-CD45: PE-Cy7 Mouse Anti-Human CD45, BD Biosciences, catalog #557748, Clone HI30 (RUO), Lots #4198943, #7313763<br>http://www.bdbiosciences.com/eu/applications/research/stem-cell-research/cancer-research/human/pe-cy7-mouse-anti-human-cd45-hi30/p/557748<br>PE-CD18: PE Mouse Anti-Human CD18, BioLegend, catalog #302108, Clone TS1/18, Lot #B194455<br>https://www.biolegend.com/en-us/products/pe-anti-human-cd18-antibody-849
Validation	All antibodies were obtained from commercial sources where the manufacturer has done appropriate validations. The manufacturer websites are provided with the antibody information. These antibodies have been used in multiple publications and by our group previously.

Eukaryotic cell lines

Policy information about cell lines

Cell line source(s)	All cell lines originate from ATCC or are derivatives of ATCC lines, except for EBV immortalized B cells (developed by the Clinical Immunology Core Laboratory at Cincinnati Children's Hospital as a fee for service, and EBV immortalized B cells from a LAD patient (that were obtained as described in the methods). EJ5-GFP NHEJ reporter cell line was obtained as described in the methods.
Authentication	No formal authentication was performed. However, we believe these lines are authentic, as we obtain lines from ATCC and freeze down stocks of 20-100 vials of early passage, store and log them into a CFR11 compliant computer program and the inventory is traceable.
Mycoplasma contamination	The EJ5-GFP HeLa were tested for mycoplasma.
Commonly misidentified lines<br>(See ICLAC register)	N/A

Flow Cytometry

Plots

Confirm that:

☒ The axis labels state the marker and fluorochrome used (e.g. CD4-FITC).

☒ The axis scales are clearly visible. Include numbers along axes only for bottom left plot of group (a 'group' is an analysis of identical markers).

☒ All plots are contour plots with outliers or pseudocolor plots.

☒ A numerical value for number of cells or percentage (with statistics) is provided.

Methodology

Sample preparation	For assessment of HDR by flow cytometry, live cells were harvested and transferred into tubes and washed once with 1XPBS. If antibody staining was required, cells were resuspended in 1X PBS plus the appropriate dilution of antibody and incubated at 4°C for at least 20 minutes. After staining, cells were again washed in 1X PBS and then resuspended in 1X PBS for analysis by flow cytometry.
Instrument	For analysis: BD FACS Canto, BD LSR II; For cell sorting: BD FACSAria II
Software	BD FACSDiva Version 8.0.1 for collection and analysis.
Cell population abundance	Sorted single cells for HDR confirmation were cloned and subjected to qPCR for HDR and GFP.
Gating strategy	For the viability experiment in Jurkat and K562 cells with eFluor 780 fixable viability dye, reference figure S7 for gating strategy.<br>For CD45-GFP donor experiments, reference figure S13a,b for gating strategy.<br>For AAVS1-CD18 donor experiments, reference figure 4a for gating strategy.<br>For CCR5-GFP donor experiments, reference figure S11a for gating strategy.<br>For AAVS1-GFP donor experiments, reference figure S10a,b for gating strategy.

☒ Tick this box to confirm that a figure exemplifying the gating strategy is provided in the Supplementary Information.
